# On the role of monetary incentives in risk preference elicitation experiments

**DOI:** 10.1007/s11166-022-09377-w

**Published:** 2022-04-20

**Authors:** Andreas Hackethal, Michael Kirchler, Christine Laudenbach, Michael Razen, Annika Weber

**Affiliations:** 1 Goethe University Frankfurt and Leibniz Institute for Financial Research SAFE, Frankfurt, Germany; 2 University of Innsbruck, Innsbruck, Austria; 3 University of Bonn, Bonn, Germany

**Keywords:** Risk preferences, Incentives, Experimental economics, Risk aversion, C91, D01, D81

## Abstract

Incentivized experiments in which individuals receive monetary rewards according to the outcomes of their decisions are regarded as the gold standard for preference elicitation in experimental economics. These task-related real payments are considered necessary to reveal subjects’ “true preferences.” Using a systematic, large-sample approach with three subject pools of private investors, professional investors, and students, we test the effect of task-related monetary incentives on risk preferences in four standard experimental tasks. We find no significant differences in behavior between and within subjects in the incentivized and non-incentivized regimes. We discuss implications for academic research and forions in the field.

**Supplementary Information:**

The online version contains supplementary material available at 10.1007/s11166-022-09377-w.

Risk is inherent to economic decision-making across many real-life domains, such as investments, health behaviors, or labor supply. As risk preferences are a fundamental determinant of decisions under risk, understanding how individuals’ preferences feed into decisions is essential to the study of individual decision-making. As a result, assumptions about individuals’ attitudes toward risk are central ingredients in many seminal models in economics and finance e.g. (Markowitz, 1952; Merton, 1969; Pratt, 1964; Barberis et al., 2001; Kahneman & Tversky, 1979). Researchers in the decision sciences, such as economics, finance, and neuroscience, commonly use controlled experiments to assess individuals’ willingness to take risks. Experiments are often incentivized, which means that individuals receive *real monetary rewards dependent on the outcome of their decisions*. The rationale behind this procedure is the assumption that individuals reveal their true preferences only if the experimental tasks have salient monetary consequences (Smith, 1976; Harrison, 1994). This practice of using incentives contrasts with practices in other social sciences, most prominently psychology, where non-incentivized, hypothetical choices are common (Camerer & Hogarth, 1999; Hertwig & Ortmann, 2001).

In this paper, we are interested in the role of incentives in the context of risk preference-elicitation tasks in economics. In many experiments, these preferences are either the main variable of interest, or serve as a control variable or a model ingredient (e.g., Kamas and Preston (2012); Alan and Ertac (2018); Saccardo et al. (2018); Thunstroem and Ritten (2019)). Incentivizing these tasks does not only induce substantial (additional) monetary costs – effectively limiting sample sizes – but also increases administrative efforts (Dohmen et al., 2011). Moreover, complicated payoff formulas may unduly increase the complexity of the experimental design and arguably make choices less realistic to subjects (Read, 2005; Bardsley et al., 2020). The rise of online surveys has facilitated the recruitment of subjects on a large scale and opened the possibility of studying choices and preferences among subject pools other than students. Obtaining accurate measures of risk attitudes of non-standard subjects such as private and professional investors is essential to understand their financial behaviors and to gauge their impact on asset prices and the macroeconomy (Guiso & Sodini, 2013). However, using task-related incentives may not always be feasible in these settings.

Against this background, we use a systematic, large-scale approach to study the impact of task-related monetary incentives on experimentally elicited risk-preference measures. Our data set is unique by combining the following important dimensions: (i) we consider four standard experimental tasks instead of focusing on a single task and (ii) we study the choices of private and professional investors in addition to the choices of students. In total, we administer an online experiment of 1,480 participants, among them 821 private investors at a large German bank, 244 professional investors at various financial companies in the EU, and 415 students at the University of Innsbruck. The experimental tasks we consider are the *staircase procedure* by Falk et al. (2016, 2018), the *gamble-choice task* by Eckel and Grossman (2002), the *paired lottery choice task* by Holt and Laury (2002), and the *investment game* by Gneezy and Potters (1997). We randomly assign subjects to two incentive conditions. Respondents in the $\textsc {flat}$ condition receive a fixed fee as a reward for participation. Respondents in the $\textsc {incentives}$ condition – in addition to the fixed participation fee – receive a task-related payment according to the outcome of their decision in one randomly selected experimental task.

In 10 of the 12 in-sample mean comparisons in the main experiment, we find no significant differences between the participants’ choices in the $\textsc {flat}$ and the $\textsc {incentives}$ condition. Only for the Holt and Laury (2002) elicitation task, we document a small increase in risk aversion for students and professional investors with incentives. Also, the standard deviations and distributions of individuals’ choices in each task by incentive condition do not differ significantly for all comparisons with the only exception being the distribution of switching points in the Holt and Laury (2002) task in the professional investor sample. The propensity to provide an extreme response does not significantly differ by incentive condition across all three subject pools. Moreover, we also find no significant differences by incentive condition with respect to other aspects of decision quality, such as effort provided. Task-specific response times are similar and while drop-out rates in the experiment differ considerably across subject pools, results reveal no differences in the propensity to drop out by incentive condition.

In two extensions with student samples, we (i) examine the role of incentives within subjects by running the respective other incentive condition six months later and (ii) add a third treatment where subjects receive a fixed participation fee equal to the average payout in the $\textsc {incentives}$ condition. We are (i) able to confirm our results also in the within-subject analysis, where we again find no significant differences when comparing the average choices subjects made in the $\textsc {incentives}$ to their choices in the $\textsc {flat}$ condition in all four experimental tasks. We also do not find evidence for order effects, which may occur in a within-subject setting. Concerning the payoff, we (ii) find that the absolute level of the fixed participation fee does not alter our results. In particular, we show that student subjects behave virtually identically under two fixed participation fee conditions that vary in payout by a factor of three.

Our results complement prior research investigating hypothetical bias in decisions under risk. Studies of whether and how task-related incentives affect subjects’ risk-taking in economic experiments have produced mixed results. While some investigations find that subjects’ behavior is more risk averse when choices have real consequences (Holt & Laury, 2002, 2005; Harrison et al., 2005), other studies find no differences in subjects’ choices across incentive conditions (Beattie & Loomes, 1997; Kuehberger et al., 2002; Gneezy et al., 2015).^1^ Furthermore, Smith and Walker (1993) and Camerer and Hogarth (1999) find that incentives lessen the variance of experimental measures. They argue that incentives might help to reduce instances of extreme outliers caused by otherwise inattentive or unmotivated subjects. Comparing real and hypothetical decisions, Camerer and Mobbs (2017) observe differences in brain activity, although not in all of the decision domains studied.^2^ More recently, Etchart-Vincent and l’Haridon (2011) document that differences in the incentive scheme have no effect in the loss domain, whereas incentives matter for risk-taking in the gain domain. We contribute to this literature by providing a *comprehensive picture* of the role of incentives in the experimental elicitation of risk preferences. Rather than focusing on a single experimental task, we run a battery of commonly used risk-elicitation experiments to asses potential effects of incentivization on both location and dispersion. Importantly, with close to 1,500 respondents, our study is high-powered and allows for conclusive inferences even in the case of null results. While we cannot make statements about whether our findings also hold in very complex and time-consuming experiments (e.g., at the end of a 2-hour session) or for high stake sizes, we consider our deliberate focus on low and moderate stake sizes an advantage, as these are a common standard in the literature and increase the generalizability of our study for state-of-the-art procedures.^3^

Second, we add to the literature on the generalizability of findings obtained in laboratory experiments with standard student subjects e.g., (List, 2003; Haigh & List, 2005; Alevy et al., 2007). In general, existing studies find substantial variation in (risk) preferences across, but also within countries, which suggests that individual characteristics play an important role e.g., (Falk et al., 2018). Given that these characteristics matter for the preferences per se, they may also matter in the responsiveness to incentives in the elicitation process. Besides examining the role of incentives in experiments with students as subjects, we run our experiments with two large, non-standard subject pools of private and professional investors. Given the ramifications for asset prices and the macroeconomy, obtaining valid measures of these subjects’ risk preferences – and the interplay with task-related incentives – is of great interest to academics, regulators, and policy makers.

## Experimental design

### Experimental tasks

We consider four of the most widely used experimental tasks for eliciting risk preferences, which we introduce below.^4^ Table 1 provides details on the parameterization of the gambles involved in each of the tasks. The experimental instructions are provided in Online Appendix D. The euro amounts stated refer to the payoff parameters in the private and professional investor sample. To align stake sizes for all subject pools with the standards in the experimental economics literature, we divide these euro amounts by four to obtain corresponding payoff profiles for the student sample see, e.g., (Haigh & List, 2005; Kirchler et al., 2018).

**Table 1:** Overview of risky choices in the single tasks

**Task**	**Choice(s)**		
**FA**	Sequence of four choices between a fixed amount A and a 50/50 gamble B		
	Choice	*Option A*	*Option B*
	(1)	€24 with *p *= 1	€45 with *p* = 0.5; €0 with *p* = 0.5
	(2) if (1) = A	€12 with *p *= 1	€45 with *p* = 0.5; €0 with *p* = 0.5
	(2) if (1) = B	€36 with *p *= 1	€45 with *p* = 0.5; €0 with *p* = 0.5
	...	...	...
	*See Fig. *A1* in Online Appendix *A* for all conditional sequences of choices.*		
**EG**	Choice of preferred lottery from a menu of six 50/50 gambles		
	*Option 1*	€21 with *p* = 0.5; €21 with *p* = 0.5	
	*Option 2*	€27 with *p* = 0.5; €18 with *p* = 0.5	
	*Option 3*	€33 with *p* = 0.5; €15 with *p* = 0.5	
	*Option 4*	€39 with *p* = 0.5; €12 with *p* = 0.5	
	*Option 5*	€45 with *p* = 0.5; €9 with *p* = 0.5	
	*Option 6*	€52 with *p* = 0.5; €2 with *p* = 0.5	
**HL**	Ten separate choices between two lotteries A and B		
	Choice	*Option A*	*Option B*
	(1)	€24 with *p* = 0.1; €19.20 with *p* = 0.9	€46.40 with *p* = 0.1; €1.20 with *p* = 0.9
	(2)	€24 with *p* = 0.2; €19.20 with *p* = 0.8	€46.40 with *p* = 0.2; €1.20 with *p* = 0.8
	(3)	€24 with *p* = 0.3; €19.20 with *p* = 0.7	€46.40 with *p* = 0.3; €1.20 with *p* = 0.7
	(4)	€24 with *p* = 0.4; €19.20 with *p* = 0.6	€46.40 with *p* = 0.4; €1.20 with *p* = 0.6
	(5)	€24 with *p* = 0.5; €19.20 with *p* = 0.5	€46.40 with *p* = 0.5; €1.20 with *p* = 0.5
	(6)	€24 with *p* = 0.6; €19.20 with *p* = 0.4	€46.40 with *p* = 0.6; €1.20 with *p* = 0.4
	(7)	€24 with *p* = 0.7; €19.20 with *p* = 0.3	€46.40 with *p* = 0.7; €1.20 with *p* = 0.3
	(8)	€24 with *p* = 0.8; €19.20 with *p* = 0.2	€46.40 with *p* = 0.8; €1.20 with *p* = 0.2
	(9)	€24 with *p* = 0.9; €19.20 with *p* = 0.1	€46.40 with *p* = 0.9; €1.20 with *p* = 0.1
	(10)	€24 with *p* = 1	€46.40 with *p* = 1
**GP**	Decision what fraction of €24 to invest in a project that pays 2.5 times the amount invested or 0 with equal probability.		
	€24 - € invested + 2.5 $\times$ € invested, with *p* = 0.5		
	€24 - € invested, with *p* = 0.5		

The table presents the gambles involved in the four risk preference elicitation tasks. Euro values stated refer to the parametrization in the private and professional investor sample. For subjects in the student sample, all values are divided by 4. $\textsc {FA}$ takes a value between 1 and 16, according to the certainty equivalent resulting from the last of the four choices in the staircase risk task. $\textsc {EG}$ is the rank (1–6) of the gamble chosen from a menu of six 50/50 gambles, increasing in risk. $\textsc {HL}$ is the number of decision rows left after switching to the higher-risk lottery, ranging from 0 to 10. $\textsc {GP}$ is the EUR amount invested in the risky project and takes values between 0 and 6. Investment amounts can be adjusted in steps of €2. Higher values imply higher risk tolerance across all four tasks

(i) A *staircase procedure* typically consists of a series of interdependent binary choices see (Cornsweet, 1962). We use the method by Falk et al. (2016, 2018) ($\textsc {FA}$) that aims to elicit subjects’ certainty equivalent for a given lottery in a series of decisions. A similar approach has already been used by Barsky et al. (1997). We ask subjects to choose between a lottery paying €45 or €0 with equal probability, and a safe payment of €24. Subjects who prefer the lottery in the first stage are offered a higher safe payment (€36) in the second decision, whereas subjects who prefer the safe payment are presented a lower safe payment (€12). The payoffs of the lottery remain constant across the decision rounds. In our specification, the payout of the safe alternative varies from €3 to €45. After four decision rounds, the staircase design allows the researcher to pin down a narrow interval for subjects’ certainty equivalent as a measure of their risk preference, with certainty equivalents being higher for more risk-tolerant individuals. We provide an exposition of the entire sequence of decisions in the four decision rounds in Fig. A1.
(ii) The *gamble-choice task* by Eckel and Grossman (2002) ($\textsc {EG}$) asks subjects to choose their preferred lottery specification from a menu of six 50/50 gambles (see Table 1). The first lottery offers a secure payoff of €21 in both states of the world. Subsequently, the difference between the two possible payoffs widens, as the first payoff increases by €6 while the second payoff decreases by €3 in each subsequent lottery. Consequently, the rank of the lottery chosen, ranging from 1 to 6, serves as a measure of a subject’s risk tolerance. Subjects with higher risk tolerance will choose lotteries farther down the list, as these offer higher expected returns at higher levels of risk.
(iii) The *paired lottery choice task* by Holt and Laury (2002) ($\textsc {HL}$) presents subjects with 10 separate decisions between a lottery A that pays either €24.00 or €19.20 and a lottery B that pays either €46.20 or €1.20 (see Table 1). In the first decision, the probability for the high [low] state is 10% [90%] in both lotteries. In each subsequent decision, the probability of the high [low] state increases [decreases] by 10%. Hence, in each decision, choosing lottery A is less risky than choosing lottery B. At the same time, however, the expected value of lottery A increases from €19.68 to €24.00, while the expected value of lottery B increases from €5.70 to €46.20. As a measure of individuals’ willingness to incur risk, we mark the decision where a subject switches from lottery A to lottery B. Subjects with higher risk tolerance will switch earlier to lottery B.^5^ For ease of interpretation, we count the number of rows after the switching point, such that higher values imply higher risk tolerance.^6^
(iv) The *investment game* by Gneezy and Potters (1997) ($\textsc {GP}$) stylizes an investment decision. In this task, subjects receive an initial endowment of €24 and are asked to decide which fraction to invest in a project that pays either 2.5 times the invested amount or €0, with equal probability (see Table 1).^7^ The amount not invested is kept in either state of the world. As is apparent from the parametrization, higher investments increase both expected value and variance of the payoff. We use the amount a subject invests in the risky project as a measure of risk tolerance such that higher values indicate higher levels of risk tolerance. Risk-neutral and risk-seeking subjects will invest their entire endowment.

### Treatments and payment of subjects

We randomly assign subjects to one of the two treatments in a between-subjects design. In the $\textsc {flat}$ condition, subjects receive a fixed fee as a reward for participating in the experiment. The fixed participation fee is €12 for subjects in the private and professional investor samples, and is €3 in the student sample. Respondents are explicitly informed that they will be asked to choose among several options with different *hypothetical* payoff profiles and that the payoffs resulting from their decisions will not actually be paid out. Payment of the fixed participation fee is independent of the choices made in the experiment. In addition to the participation fee, subjects in the $\textsc {incentives}$ condition are paid the earnings resulting from their choice in one experimental task, which is randomly selected at the end of the experiment. In case the selected task involves a series of decisions, a second random draw determines the decision to be paid out. Subjects are then paid according to their choice and the random outcome of the respective lottery. Subjects in both conditions are presented with the same experimental tasks and experimental instructions (except for minor differences necessary to explain the payment protocols).

### Experimental protocol

To determine the target number of subjects to be recruited, we performed a power analysis following Cohen (1988) for behavioral sciences. We aimed to maximize statistical power for each sample given the particular constraints with respect to recruitment possibilities. Applying the predefined target parameters to our realized sample sizes, our tests have $90\%$-*a priori* power to detect effect sizes as low as 0.23, 0.42, and 0.32 in mean differences between the $\textsc {incentives}$ and $\textsc {flat}$ condition for the sample of private investors, professional investors, and students, respectively. These numbers are in the range of small and small-to-medium effect sizes, as suggested by Cohen (1988). We provide details on our power analysis in Table C1 in Online Appendix C.

The experiment was administered online using Limesurvey. We recruited subjects for participation via e-mail. Through e-mail, we recruited student participants from the University of Innsbruck using Hroot (Bock et al., 2014). In addition, we invited private investors from a panel of 2,000 clients of a large German brick-and-mortar bank who regularly participate in short online surveys/experiments administered by Goethe University Frankfurt. Third, we recruited professional investors via two channels. Two-thirds of the professional investor sample were recruited from the proprietary subject pool of professional investors (http://www.before.world) at the University of Innsbruck, some of whom had participated in previous unrelated studies. The remaining professionals are fund managers from different European countries whom we identified via their fund affiliation using data from Morningstar. 

To avoid potential selection bias into either of the incentive conditions, we use a standardized invitation letter for all subjects. Subjects learn about the payment protocol relevant for them only upon starting the experiment.

### Data and sample characteristics

We collected data for our main analyses in April and May 2020.^8^ Overall, 1,512 subjects completed the experiment. Once the experiment started, we offered unlimited time to finish to avoid exerting time pressure on subjects who were potentially engaged with risk elicitation experiments for the first time. However, to screen out participants who plausibly did not take the experiment seriously and to avoid potential noise due to outliers, we drop subjects in the top (99%) and bottom percentiles (1%) of the response time.^9^ The final sample consists of 1,480 subjects, comprising 821 subjects from the private investor sample, 244 from the financial professional investor sample, and 415 from the student sample. The median response time in the final sample is 13.22 minutes with a standard deviation of 9.88 minutes.

Panel A of Table 2 describes the private investor sample. Respondents in this sample are retail clients at a large German bank with a national branch network and are part of a regular online survey panel.^10^ Of the respondents, 26 percent are female, the average age is 53 years, and private investors’ reported average net household income is €4,292. Of the private investors, 79 percent invest in stocks or stock mutual funds.

**Table 2:** Descriptive statistics

**Panel A.** Private investors					
	ALL	$\textsc {incentives}$	$\textsc {flat}$		
	Mean	Mean	Mean	*P*-value	Obs.
	(SD)				
	(1)	(2)	(3)	(4)	(5)
Female	0.25	0.28	0.23	0.102	809
	(0.44)				
Age	52.98	53.53	52.44	0.323	809
	(15.67)				
HH net income	4,250	4,151	4,347	0.288	676
	(2,402)				
Stock investor	0.79	0.79	0.79	0.949	821
	(0.41)				
Smartphone	0.19	0.18	0.19	0.750	821
	(0.39)				
Total time	16.33	17.33	15.37	0.005	821
	(10.07)				
Payoff	21.71	31.72	12.00	0.000	821
	(16.38)				
**Panel B.** Professional investors					
	ALL	$\textsc {incentives}$	$\textsc {flat}$		
	Mean	Mean	Mean	*P*-value	Obs.
	(SD)				
	(1)	(2)	(3)	(4)	(5)
Female	0.11	0.11	0.11	1.000	244
	(0.31)				
Age	41.89	42.19	41.59	0.624	244
	(9.50)				
*Job position*					
- Fund manager	0.35	0.37	0.34	0.594	244
- Portfolio manager	0.18	0.15	0.20	0.241	244
- Analyst	0.10	0.09	0.11	0.528	244
- Risk manager	0.07	0.08	0.06	0.453	244
Smartphone	0.05	0.03	0.07	0.238	244
	(0.22)				
Total time	20.46	22.77	18.15	0.003	244
	(12.41)				
Payoff	21.72	31.43	12.00	0.000	244
	(17.17)				
**Panel C.** Students					
	ALL	$\textsc {incentives}$	$\textsc {flat}$		
	Mean	Mean	Mean	*P*-value	Obs.
	(SD)				
	(1)	(2)	(3)	(4)	(5)
Female	0.57	0.58	0.54	0.159	415
	(0.50)				
Age	23.94	24.16	23.71	0.220	415
	(3.22)				
Net income	748.88	727.14	772.54	0.167	403
	(370.51)				
Stock investor	0.25	0.23	0.26	0.480	415
	(0.43)				
Smartphone	0.04	0.03	0.04	0.551	415
	(0.19)				
Total time	13.45	14.35	12.52	0.026	415
	(8.34)				
Payoff	5.38	7.67	3.00	0.000	415
	(4.02)				

Panel A shows summary statistics for the 821 respondents in the private investor sample. Panel B shows summary statistics for the 244 respondents in the finance professionals sample. Panel C shows summary statistics for the 628 participants in the student sample. Information of respondents’ household net income is only available for 676 of the 821 respondents in the private investor sample, due to non-response. Similarly, 12 of the 415 students choose “prefer not to answer”. Note that private investor demographics have been elicited in an earlier survey wave and were not re-elicited for the purpose of our study. This causes demographics to be missing for a number of private investors who did not participate in this earlier survey. Stock investor is an indicator equal to one for participants who invest in stocks or stock mutual funds. Smartphone is an indicator of whether the respondent has participated in the experiment using a smartphone. Total time is the time (in minutes) a subject has spend to complete the entire experiment. Payoff is the final payoff participants receive after completing the experiment. It is fixed in the $\textsc {flat}$ conditions. For subjects in the $\textsc {incentives}$ condition, it depends on the choice and resulting outcome in one randomly determined experimental task. Task-related payoffs in the $\textsc {incentives}$ condition are in addition to the fixed participation fee paid to subjects in the $\textsc {flat}$ condition. Potential payoffs in the student sample result from dividing payoff options presented to private investors and finance professionals by 4. Column 4 reports *p*-values from a two-sided t-test of equal means between subjects in the $\textsc {incentives}$ and $\textsc {flat}$ condition

The private investor sample is well balanced along these characteristics across the two treatment arms, as indicated by the close averages and corresponding high *p*-values from a two-sided t-test of equal means in column 4 of Table 2 (Panel A). The only significant difference between subjects in the various incentive conditions arises in overall response time, which is significantly higher under the $\textsc {incentives}$ condition. However, as we show below, this difference does not result from longer decision times in the single experimental tasks in the $\textsc {incentives}$ condition, but is explained by subjects’ need to spend more time reading longer texts outlining the payoff protocol in the $\textsc {incentives}$ condition. This condition applies equally to all three subsamples.

Panel B of Table 2 describes the professional investors sample. Respondents in this sample are predominantly male (89 percent) and the average age is 42 years. The majority of professional investors are fund managers (35 percent), portfolio managers (18 percent), analysts (10 percent), and risk managers (7 percent). Again, the sample is well balanced across both treatment arms, as indicated by the close averages and corresponding high *p*-values from a two-sided t-test in column 4 of Table 2 (Panel B) in the Online Appendix.

Panel C of Table 2 characterizes the student sample. Most subjects in the student sample are female (57 percent), and the average age is 24 years. One in four student subjects invests in stocks or stock mutual funds. Both treatments in our student sample are well balanced along these characteristics.

## Results

### Risk-taking

***Result 1:*** *Risk-taking of private investors, professional investors, and students does not differ across incentive conditions in three of the four experimental tasks. In the* $\textsc {HL}$ *task, professional investors and students engage in slightly less risk-taking in the* $\textsc {incentives}$ *than in the* $\textsc {flat}$ *condition*.

**Fig. 1:**
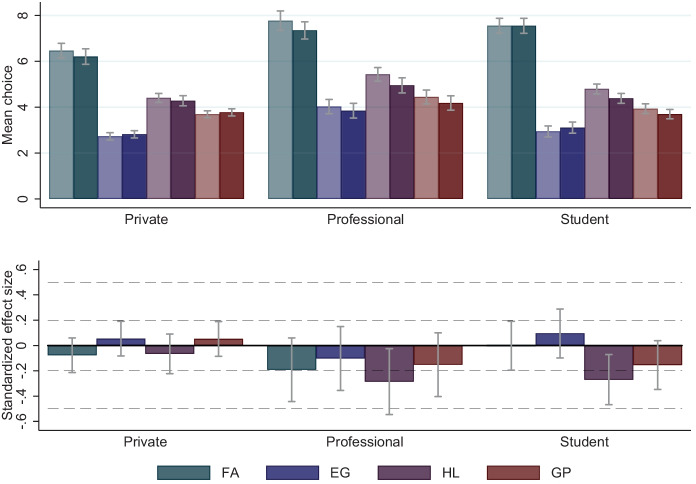
Effect of task-related incentives on risk-taking. *Notes:* Upper panel: Mean choices by subject pool (3 blocks), task (4 colors), and incentive condition (2 shades). The light (dark) shaded bars represent the choices of subjects in the $\textsc {flat}$ ($\textsc {incentives}$) condition. For all tasks, higher levels indicate greater risk-taking. Owing to the different nature of the underlying tasks, the absolute height of the bars cannot be compared across tasks. $\textsc {FA}$ takes a value between 1 and 16, according to the ordinal rank of the certainty equivalent resulting from the last of the four choices in the staircase risk task. $\textsc {EG}$ is the rank (1-6) of the gamble chosen from a menu of six 50/50 gambles, increasing in risk. $\textsc {HL}$ is the number of decision rows left after switching to the higher-risk lottery, ranging from 0 to 10. $\textsc {GP}$ is the euro amount invested in the risky project and takes values between 0 and 24 for private and professional investors, and values between 0 and 6 for students. For illustration purposes in the above graph, we align these values across samples by dividing the invested amount in the private and professional investor sample by 4. Lower panel: Standardized treatment effects of incentivization on risk-taking, by subject pool and task. Bars represent the coefficient of an indicator variable for whether a subject has been assigned to the $\textsc {incentives}$ condition in regressions with standardized choices. We standardize choices by deducting the mean and dividing by the standard deviation in the respective subject pool. Error bars indicate 95%-confidence intervals

Support: We start our analysis by comparing mean choices in the four experimental tasks between subjects in the $\textsc {flat}$ and $\textsc {incentives}$ condition. The upper panel in Fig. 1 displays average choices by experimental task, incentive condition, and subject pool. Throughout, higher values (higher bars) indicate higher levels of risk tolerance. For $\textsc {FA}$, we display a value between 1 and 16 according to ordinal ranking of the resulting certainty equivalent. For $\textsc {EG}$, we display the rank (1 to 6) of the gamble chosen from the menu of six 50/50 gambles. In the $\textsc {HL}$-task, we present the number of decision rows left after switching to the higher-risk lottery, ranging from 0 to 10. For $\textsc {GP}$, we show the euro amount invested in the risky investment. To make choices comparable across subject pools, we divide the amount invested in the $\textsc {GP}$-task in the private and professional investor subsamples by four. $\textsc {GP}$ hence takes on values between 1 and 6.^11^ The light (dark) shaded bars represent subjects’ choices in the $\textsc {flat}$ ($\textsc {incentives}$) condition. We report the *p*-values of two-sided t-tests for equality of mean choices in Table A4 in the Online Appendix.^12^

We find no significant differences between subjects’ choices in the various incentive conditions in three of the four experimental tasks (see Fig. 1). In the case of $\textsc {HL}$, we find that subjects’ behavior is slightly more risk-averse in the $\textsc {incentives}$ condition than under the $\textsc {flat}$ regime in the student sample (6.21 vs. 6.62, t-test, *p*-value = 0.008, *N* = 388) and the professional investor sample (6.05 vs. 5.57, t-test, *p*-value = 0.032, *N* = 226). In the lower panel of Fig. 1, we display standardized treatment effects sizes, along with 95% confidence intervals. Within each subject pool, we standardize the choices in the different tasks by subtracting the mean and dividing by the standard deviation of the distribution of choices in the respective subject pool. We then regress standardized choices on an indicator variable for whether a subject has been assigned to the $\textsc {incentives}$ condition. The figure confirms the above finding and illustrates the relative magnitude of the decrease in risk-taking in the $\textsc {HL}$ task on the part of finance professionals and students in the $\textsc {incentives}$ condition. In both cases, the difference amounts to 0.27 of a standard deviation.

Interestingly, we observe that private investors exhibit a systemically lower risk tolerance than professional investors.^13^ The differences between these two groups, which we document in Table A1 in the Online Appendix, are significant at the 5%- level (pairwise t-tests) for all tasks in both treatments. For instance, private investors, on average, invest around 10% less than professional investors in the risky project in the incentivized $\textsc {GP}$ task.

In addition to comparing the means, we also compare variances and distributions of individuals’ choices in each task by incentive condition, separately for each subject pool (see Table A2 and Fig. A2 in the Online Appendix).^14^ We find that F-Tests fail to reject the null of equal standard deviations under the $\textsc {incentives}$ and the $\textsc {flat}$ condition, respectively, for all comparisons. Further, Kolmogorov-Smirnov tests fail to reject the null hypothesis of equal distributions under both incentive regimes across tasks and subject pools, with the only exception being the distributions of the switching point in the $\textsc {HL}$ task in the professional investor sample (KS-test, *p*-value = 0.022, *N *= 226; *p*-values of KS-tests are reported in Table A1 in the Online Appendix).

### Incentives and effort

***Result 2a:*** *In general, effort – as measured by decision times of subjects – does not differ across incentive conditions, tasks, and subject pools. Moreover, while drop-out rates in the experiment differ considerably across subject pools, results reveal no significant differences in the propensity to drop out by incentive condition.*

**Fig. 2:**
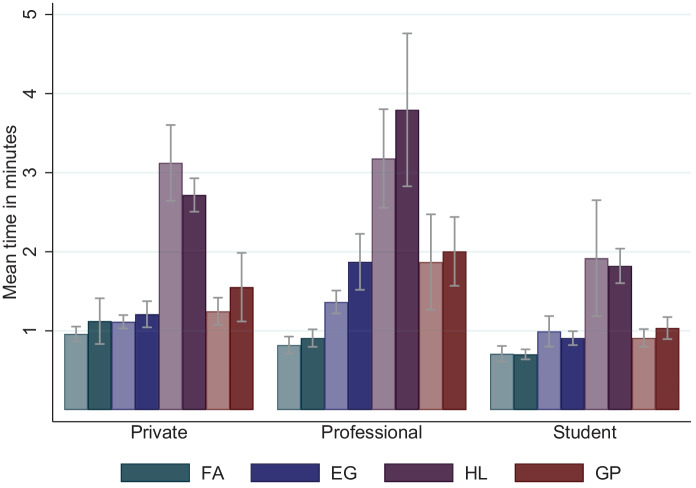
Effect of task-related incentives on decision times. *Notes*: The figure compares the average decision times in minutes for the four experimental tasks (4 colors) by incentive condition (2 shades) separately for the three subject pools (3 blocks). The light (dark) shaded bars represent the choices of subjects in the $\textsc {flat}$ ($\textsc {incentives}$) condition. Error bars indicate 95% confidence intervals

Support: Another dimension along which task-related incentives may affect subjects’ behavior in the experiments is the effort subjects apply in making decisions. We follow the literature in using decision time when making their choices as a measure of effort (Wilcox, 1993; Camerer & Hogarth, 1999) and we compare decision times in the four tasks by incentive condition. Subjects take on average 0.88 minutes to complete the $\textsc {FA}$, 1.13 minutes for the $\textsc {EG}$, 2.58 minutes for the $\textsc {HL}$, and 1.29 minutes for the $\textsc {GP}$ task. As illustrated in Fig. 2, we find no significant differences in decision times by incentive condition across tasks and subject pools, except for professional investors in the $\textsc {incentives}$ condition of the $\textsc {EG}$ task (t-test, *p*-value = 0.01, *N* = 244; see Table A3 in the Online Appendix). If we control for outliers, differences also get insignificant for that subsample. These results imply that the significantly higher *total* time spent in the experiment observed for subjects in the $\textsc {incentives}$ condition across the three subject pools (see Table 2 in the Online Appendix) results from subjects taking more time to read the details of the task-related payoff protocol rather than from spending more time thinking about their choices.

**Fig. 3:**
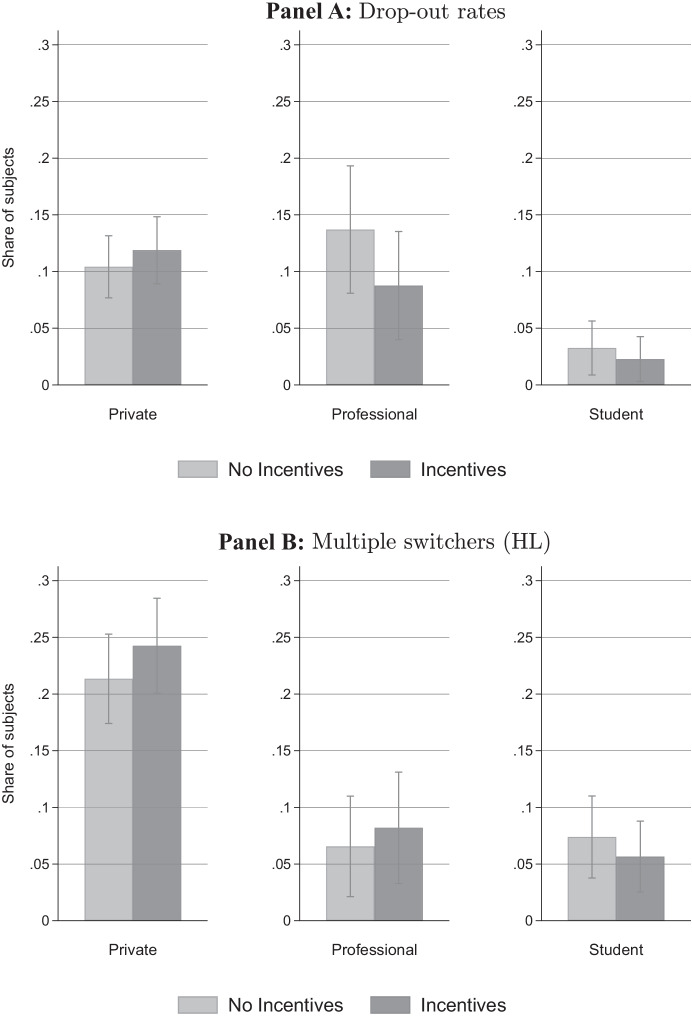
Drop-out rates and inconsistent choices. *Notes*: The figure shows the propensity of respondents to drop out from the experiment (Panel A) and the share of subjects with inconsistent answers (multiple switching points) in the HL task (Panel B) by incentive condition, separately for the three subject pools. Drop out rates are calculated based on the overall number of respondents who started the experiment (*N* = 1,882), of which 1,727 completed it. Error bars indicate 95%-confidence intervals

As a second proxy for individuals’ effort, we test whether task-related incentives act to increase subjects’ perseverance, reducing the number of participants who exit the experiment prior to completion. Overall, 1,661 subjects started the experiment, of which 1,512 completed it (and 1,480 constitute the final data set after the winsorizing procedure outlined above), translating into a drop-out rate of 9 percent. We find that drop-out rates differ considerably across subject pools and are substantially lower in the student sample (below 3 percent) than in the private investor and professional investor samples (about 11 percent in both samples). However, in all subject pools, we do not find significant differences in the propensity to drop out from the experiment across incentive conditions (see Panel A of Fig. 3 and Table A3 in the Online Appendix).

Finally, we test whether task-related incentives reduce instances of inconsistent behavior in the arguably complex $\textsc {HL}$ task. In the $\textsc {HL}$ task, we allow participants to switch between options A and B as they move down the 10 decision rows. This opportunity may result in inconsistent behavior as subjects could switch between options more than once.^15^ Across all subject pools, 15 percent of participants make inconsistent choices, which compares rather favorably to other studies (Crosetto & Filippin, 2016; Charness et al., 2013). The prevalence of inconsistent choices differs greatly across subject pools (see Panel B of Fig. 3 and Table A3 in the Online Appendix). While the choices of about 7 percent of subjects in the student and professional investor sample produce multiple switching points, 23 percent of respondents in the private investor sample show this kind of behavior. One obvious reason lies in different education levels among the three subject pools. We find the propensity to give inconsistent answers in the private investor sample to be negatively correlated with educational achievement and financial literacy. Among private investors who have completed a college degree (*N *= 401), the share of respondents giving inconsistent answers amounts to 13 percent. Accordingly, Dave et al. (2010) show that less sophisticated subjects have trouble understanding the $\textsc {HL}$ protocol. Importantly, we find no evidence that the incentive regime does affect the propensity to behave inconsistently in the $\textsc {HL}$ task across the three subject pools.^16^

***Result 2b:*** *In general, seriousness of subjects – as measured by variation in decisions – does not differ across incentive conditions.*

Support: Smith and Walker (1993) and Camerer and Hogarth (1999) argue that incentives might help to reduce instances of extreme outliers caused by otherwise inattentive or unmotivated subjects and hence lead to lower variance. From a methodological point of view, incentives could thus contribute to higher-quality data and improve statistical power (Camerer & Hogarth, 1999). We test this conjecture by pairwise comparisons of standard deviations across treatments (Table A2). Our results reveal no significant differences between incentivized and non-incentivized tasks ($p>0.05$ for all comparisons). In fact, standard deviations vary much more across samples than across treatments. Using dispersion of results as an indicator for the effort subjects put in answering the risk elicitation tasks, we thus cannot confirm that incentives increase subject commitment.

Finally, we compare the probability of extreme choices for each task by incentive condition. To do so, we define an indicator equal to one if a respondent selects into the lowest or highest risk tolerance category, according to the respective measure. In Fig. A3 in the Online Appendix, we illustrate that the propensity to provide an extreme response does not significantly differ by incentive condition at conventional significance levels of 5 percent or higher across all three subject pools, according to two-sided t-tests (see Table A4 in the Online Appendix).

### Incentives and consistency in risk-taking across tasks

***Result 3:*** *As measured by the standard deviation of an individual’s standardized choices, intra-subject consistency of risk-taking across the four experimental tasks is unaffected by the incentive condition.*

**Fig. 4:**
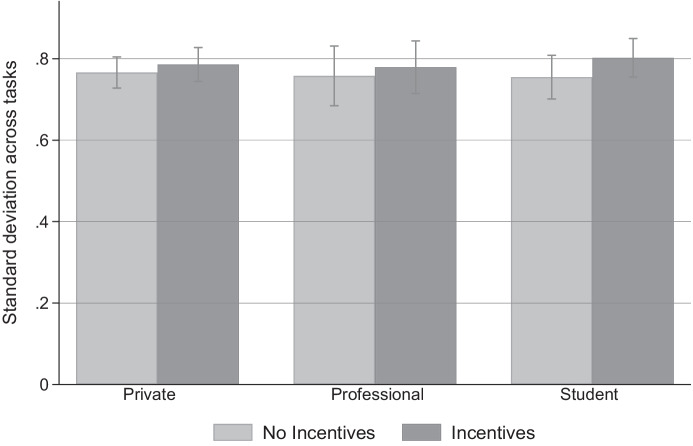
Within-subject consistency in risk-taking across tasks. *Notes*: The figure compares the mean within-subject standard deviation for the four experimental tasks by incentive condition separately for the three subject pools. We standardize choices in the single tasks by deducting the mean and dividing by the standard deviation of choices made in the given task in the relevant subject pool. We then calculate the within-subject standard deviation over a subject’s four standardized choices. The light (dark) shaded bars refer to subjects in the $\textsc {flat}$ ($\textsc {incentives}$) condition. Error bars indicate 95%-confidence intervals.

Support: We investigate whether the incentive regime affects the individual’s consistency of risk-taking (relative to their peers’ decisions) across the four experimental tasks. To calculate each subject’s standard deviation, we first standardize choices in the four experimental tasks by subtracting the mean and dividing by the standard deviation of the distribution of choices in the respective task and in the relevant subject pool. For each subject, we then compute the standard deviation over the subject’s standardized choices in the four tasks.^17^ We do not find any evidence that task-related incentives affect the intra-subject standard deviation of choices across the three subject pools (see Fig. 4 and Table A3 in the Online Appendix). Interestingly, the intra-subject standard deviation decreases strongly when excluding the $\textsc {HL}$ task from the consistency measure in general (see Fig. A4 in the Online Appendix). Singular exclusion of any of the other three choices does not produce a similar effect.

## Extensions

### Within-subject results

Until now, our experimental method was based on a between-subject design, randomly allocating subjects to either the $\textsc {incentives}$ or the $\textsc {flat}$ condition. Conditional on a random assignment between groups, the between-subject approach is perceived to be the more conservative method. Within-subject designs may, however, be favorable in environments where subjects are likely to face repeated decisions (Charness et al. (2012)). Eliciting risk preferences with and without incentives in the same experimental session may decrease incentive effects due to a consistency preferences or experimenter demand effect based on the first decision made.

**Fig. 5:**
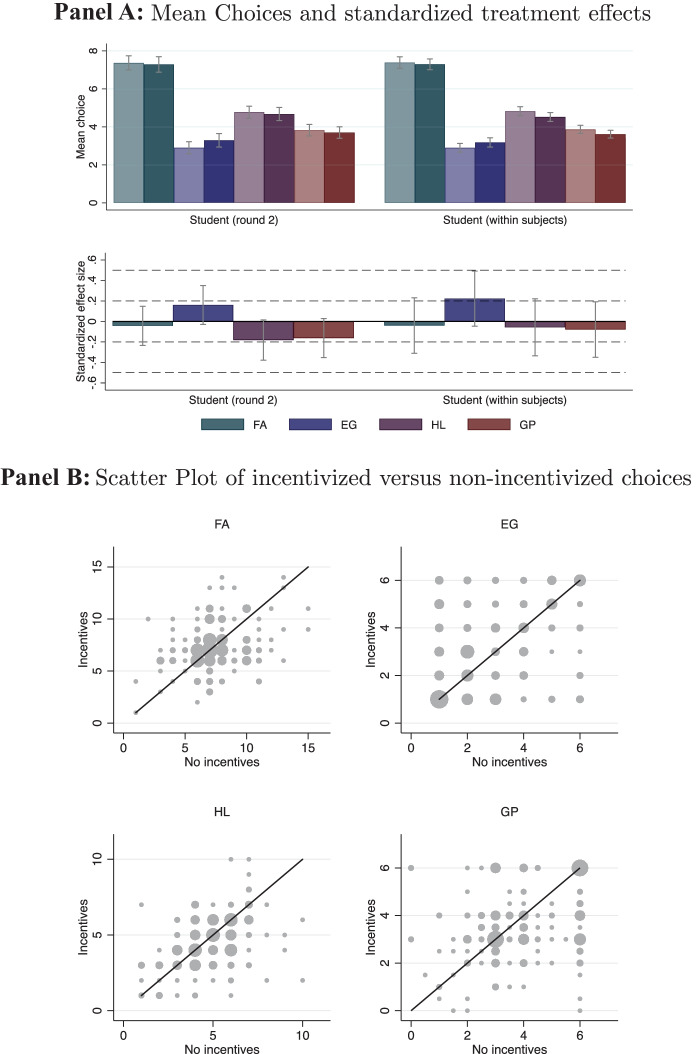
Task-related incentives and risk-taking within-subjects. *Notes*: Upper panel: Mean choices by task (4 colors) and incentive condition (2 shades) for the student sample. The light (dark) shaded bars represent the choices of subjects in the $\textsc {flat}$ as well as in the $\textsc {incentives}$ condition. The order of conditions was counterbalanced. For all tasks, higher levels indicate greater risk-taking. Owing to the different nature of the underlying tasks, the absolute height of the bars cannot be compared across tasks. $\textsc {FA}$ takes a value between 1 and 16, according to the ordinal rank of the certainty equivalent resulting from the last of the four choices in the staircase risk task. $\textsc {EG}$ is the rank (1–6) of the gamble chosen from a menu of six 50/50 gambles, increasing in risk. $\textsc {HL}$ is the number of decision rows left after switching to the higher-risk lottery, ranging from 0 to 10. $\textsc {GP}$ is the euro amount invested in the risky project and takes values between 0 and 6. Lower panel: Standardized treatment effects of incentivization on risk-taking, by task. Bars represent the coefficient of an indicator variable for the $\textsc {incentives}$ condition. We standardize choices by deducting the mean and dividing by the standard deviation. Error bars indicate 95%-confidence intervals. *Notes*: The figure presents scatter plots of the incentivized (y-axis) versus non-incentivized choices (x-axis) for the four risk tasks in the student sample weighted by the frequency of occurrence. Larger circles represent higher frequencies. The black line represents the 45 degree line. FA takes a value between 1 and 16, according to the ordinal rank of the certainty equivalent resulting from the last of the four choices in the staircase risk task. EG is the rank (1-6) of the gamble chosen from a menu of six 50/50 gambles, increasing in risk. HL is the number of decision rows left after switching to the higher-risk lottery, ranging from 0 to 10. GP is the euro amount invested in the risky project and takes values between 0 and 6

To overcome this concern, we conduct a second experiment (round) with students who participated in our first experiment (round) six months later in November 2020 see, for example, (Cavallo et al., 2017). We invited all students who participated in the first experiment and gave their consent to participate in a subsequent experiment (the second round, however, was not framed as a follow-up experiment in the narrow sense, as no references to the first round were made and also payments were administered entirely independently). Students who were previously assigned to the $\textsc {incentives}$ condition were now assigned to the $\textsc {flat}$ condition and vice versa. Overall, 213 students followed our invitation (i.e., response rate of 51.3%). For these 213 subjects, we are able to analyze the role of incentives in a within-subject design. To do so, we first replicate results from our between-subject analysis in Fig. 1 within subjects. Panel A of Fig. 5 shows that we are able to confirm our results also in the within-subject analysis. We find no significant differences if we compare the average choices subjects made in the $\textsc {incentives}$ to their choices in the $\textsc {flat}$ condition in all four experimental tasks. To better understand the heterogeneity across subjects, Panel B of Fig. 5 depicts a scatter plot of the incentivized (y-axis) versus non-incentivized choices (x-axis) weighted by the frequency of occurrence. Points on the 45 degree line represent subjects who took the same decision under both treatment conditions. Systematic effects of incentives would be reflected by an overbalance of choices above (below) the 45 degree line, reflecting increased (decreased) risk taking in the presence of incentives. The scatter plots corroborate that there are no significant incentive effects and that subjects tend towards taking the same or similar decisions under the two treatment conditions.

Finally, we also test for order effects to address potential issues with task recognition among the within sample. We do so by comparing the answers of those subjects being assigned to the $\textsc {incentives}$ condition in the first wave to those who were assigned to this condition in the second wave (analogously, we analyze order effects for the $\textsc {flat}$ condition). As average choices do not significantly differ across waves ($p>0.05$ for all pairwise comparisons), we find no evidence for order effects.

### Average payment amount

In our design, average payments are lower in the $\textsc {flat}$ condition than in the $\textsc {incentives}$ condition (see Table 2). To control for the sensitivity of our results to the *absolute amount* paid to subjects, we also include a third incentive condition in the first experimental round, $\textsc {flat\_high}$, for the student sample (i.e., students were randomly allocated to one of the three treatments). Under this regime, student subjects receive a fixed participation fee equal to the average payout of students in the $\textsc {incentives}$ condition, amounting to €9. Summary statistics for this subsample are provided in Table A5. The table shows that the samples are balanced with regard to students’ personal characteristics.

Figure A5 in the Online Appendix reproduces Fig. 1 comparing choices in the student sample by incentive condition, including the third condition $\textsc {flat\_high}$. We find that the absolute level of the fixed participation fee does not alter our results. Student subjects behave virtually identically under the $\textsc {flat\_high}$ and $\textsc {flat}$ condition, as illustrated by the rightmost set of bars. Consequently, student subjects in the $\textsc {incentives}$ condition take risks similar to those in both flat fee conditions $\textsc {flat\_high}$ or $\textsc {flat}$ in the $\textsc {FA}$, $\textsc {EG}$, and $\textsc {GP}$ tasks. Again, we cannot make statements about whether our findings hold with more extreme (much higher) stake sizes as well, given that the intention of our study is to test the role of incentives for state-of-the-art procedures in the sense of standard stake sizes.

## Conclusion

We use a systematic, large-sample approach with three subject pools of private investors, professional investors, and students, to investigate the impact of task-related monetary incentives on risk preferences, elicited in four standard experimental tasks: the *staircase procedure* by Falk et al. (2016, 2018), the *gamble-choice task* by Eckel and Grossman (2002), the *investment game* by Gneezy and Potters (1997), and the *paired lottery choice task* by Holt and Laury (2002). We find no significant differences between the choices of subjects in the different incentive conditions in 10 of the 12 in-sample comparisons across subject pools. Only in the Holt and Laury (2002) task do professional investors and students behave in a slightly more risk averse manner under a task-related incentive regime than under a regime where subjects receive a flat fee for participation. These results do not change when absolute differences in payment amounts are accounted for in the student sample. We also find no significant differences across incentive conditions with respect to task-specific response times, drop-out rates, inconsistent choice behavior, and within-subject consistency in risk-taking across tasks.

Our analyses so far remain silent as to why the $\textsc {HL}$ task produces the only significant differences between monetarily incentivized and hypothetical choices in some of the tests. Our results show that exclusion of the $\textsc {HL}$ choice increases within-subject consistency in choices across the four experimental tasks, providing evidence that individuals tend to behave “differently” in this task. Future research could tackle questions as to whether incentives matter more in complex tasks or whether incentives interact with specific features of the tasks, such as the ability to capture risk-seeking behavior, which is particularly inherent to $\textsc {HL}$, but absent in some other tasks (e.g., $\textsc {EG}$).^18^

Importantly, our results do not necessarily extend to experimental tasks other than the risk-preference elicitation tasks covered. For example, evidence on the effectiveness of incentives is mixed in valuation tasks. On the one hand, real task-related incentives have been shown to matter in valuation tasks, where subjects regularly overstate their valuation of alternatives or objects if choices are only hypothetical (List & Gallet, 2001). On the other hand, Hascher et al. (2021) recently found that unincentivized rating tasks predict choices no worse that incentivized rating tasks and significantly better than incentivized willingness-to-pay procedures. In other areas, numerous studies show that purely hypothetical tasks do overstate socially desirable behaviors in subjects, such as altruism, cooperativeness, and patience (see Camerer and Mobbs (2017) for a recent review). In these settings, incentives are an integral part of the experimental design and it is hard to doubt the necessity to incentivize these tasks (Bardsley et al., 2020). We also do not argue that experimental subjects need not be paid at all. While some people may be intrinsically motivated to participate and will respond truthfully to experimental tasks (Read, 2005), payment of a flat reward for participation plausibly increases the willingness to participate and may help reduce selection into participation. As we paid all subjects a fixed reward for participation, we can only hypothesize about the potential effects of fixed participation fees.

Given the importance of risk preferences to researchers, policymakers, and industry professionals, future research should strive to settle the current methodological issues in the elicitation of individuals’ attitudes toward risk. This obligation holds for large-scale lab-in-the-field experiments, where preferences are often elicited only as a control variable and where time and money are scarce. It also holds for applied settings, most prominently the elicitation of risk preferences as part of the financial advisory process under MiFID II (see https://www.esma.europa.eu/policy-rules/mifid-ii-and-mifir; retrieved July 1, 2020).^19^ Thus, having lean experimental protocols that produce accurate measures of individuals’ risk preferences is critical. In weighing complexity of the experimental design against the accuracy of preference measures, an important criterion is that of learning more about potential hypothetical bias in standard risk elicitation tasks. Our results imply that the degree of hypothetical bias is limited in experiments administered online to private and professional investors (with non-task-related incentives).

## Supplementary information

Below is the link to the electronic supplementary material.

Supplementary file1 (PDF 1.67 MB)

## Data Availability

The study was pre-registered, and the data and replication materials will be made public upon publication of the paper.

## Footnotes

1 For extensive reviews, see Camerer (1995); Camerer and Hogarth (1999); Camerer and Mobbs (2017); Harrison (2006).

2 Closely related, Dohmen et al. (2011) validate a non-incentivized survey item designed to measure individuals’ risk attitudes using a lottery-choice experiment and find that the general risk-attitude question strongly predicts behavior in the experiment. The survey item is also found to generate the best predictor of real-world risky behaviors.

3 Encouragingly for our study, Enke et al. (2021) show that very high incentives, increasing the standard lab stakes to more than a monthly income, have hardly any effect on bias-proneness in four classical tasks (e.g., base-rate neglect or anchoring). The authors show that in none of the tasks these high stakes come even close to de-bias subjects.

4 Since we focus on the role of incentives in the elicitation of risk preferences, we do not consider qualitative questions that are also used to assess risk preferences, but are typically not incentivized.

5 Note that in the 10th decision, the higher payoff will be paid with certainty in both lotteries, so choosing the second lottery is a dominant strategy. This result provides validation of whether subjects have understood the task (Charness et al., 2020).

6 Note that we allow participants to freely switch between options A and B as they move down the 10 decision rows. This lack of constraint allows for the possibility that individuals may behave inconsistently by choosing to switch between lotteries A and B more than once. We drop these observations from our main analyses but include a specific discussion on a potential association between incentives and the propensity to behave inconsistently in Sect. 2.

7 We we use the same variant as Charness et al. (2020). Note that in the original version of Gneezy and Potters (1997), the probabilities for receiving 2.5 times the invested amount and zero are 1/3 and 2/3, respectively.

8 The second round of data for the within-subjects analyses among the student sample was collected in November 2020. Descriptive statistics for the robustness analyses (i.e., the student samples for treatment $\textsc {flat\_high}$ and the within-sample, respectively), are provided in the online appendix.

9 Our results are robust to varying this cutoff, as well as applying the cutoff to the three subsamples individually. The propensity toward excessive response times does not systematically vary with the treatment condition. We use the same cutoffs for students in the $\textsc {flat\_high}$.

10 This panel consists of about 4,000 clients who signed up to regularly participate in online surveys for the purpose of academic research. For the present study, we selected 2,000 clients from the panel, over-sampling clients with an investment account (e.g. bonds, stocks, mutual funds) as opposed to clients holding a current account only. As private investor demographics have been elicited in an earlier survey wave, they are not re-elicited in the present study, a constraint that causes demographics to be missing for a small number of private investors who did not participate in this earlier survey.

11 Investment amounts can be adjusted in steps of €0.50 (€2.00).

12 Importantly, we refrain from measuring risk preferences by calculating risk preference parameter. As our focus is on within-task comparisons, results of risk preference parameter estimates would be very similar to what we report in this paper.

13 We refrain from directly comparing students to the other two samples due to the variation in the respective payoff profiles.

14 Panel A displays choices of private investors, and Panels B and C refer to professional investors and students, respectively. Light (dark) colored bars represent the choices of subjects in the $\textsc {flat}$ ($\textsc {incentives}$) condition.

15 Remember, a risk-averse agent should start out by choosing option A in the first decision, and then switch to option B at some point before the last decision.

16 Holt and Laury (2002) observe the number of multiple switchers to be lower in real payoff treatments, compared to hypothetical choices. They also observe less inconsistent choices in higher-payoff treatments compared to their baseline real payoff treatment, with the difference being small, however.

17 More precisely, note that this measure combines between and within elements. It shows how strongly a subject’s relative (compared to the peer sample) risk-taking varies across tasks.

18 See Crosetto and Filippin (2016) for a recent discussion of how characteristics of risk elicitation tasks such as complexity, availability of a safe option, or the range of risk attitudes may affect measured behaviors. To date, the literature contains no discussion of how these factors may interact with the presence or absence of task-related incentives.

19 More generally, several studies have tested the power of preferences using standard experimental tasks to explain individuals’ risky behaviors in the lab and in the field. In a recent study, Charness et al. (2020) show that incentivized standard measures of risk preferences are found to have very limited external validity (i.e., field behavior is measured with financial, health, and employment decisions in a representative Dutch population). In addition, those authors show that simpler measures perform better than the more complex measures.
