# Supplementary material for: On the role of monetary incentives in risk preference elicitation experiments
Source: J Risk Uncertain. 2022 Apr 20;66(2):189–213. doi: 10.1007/s11166-022-09377-w (PMC10023624; doi:10.1007/s11166-022-09377-w)
Supplement: Supplementary file 1 — Supplementary file1 (PDF 1.67 MB) [file 11166_2022_9377_MOESM1_ESM.pdf]

Online Appendix: On the role of monetary incentives in risk preference elicitation

Andreas Hackethal[12], Michael Kirchler[3], Christine Laudenbach[1],
Michael Razen[3], Annika Weber[12]

A Additional Figures

Figure A1: Sequence of choices in the staircase procedure FA

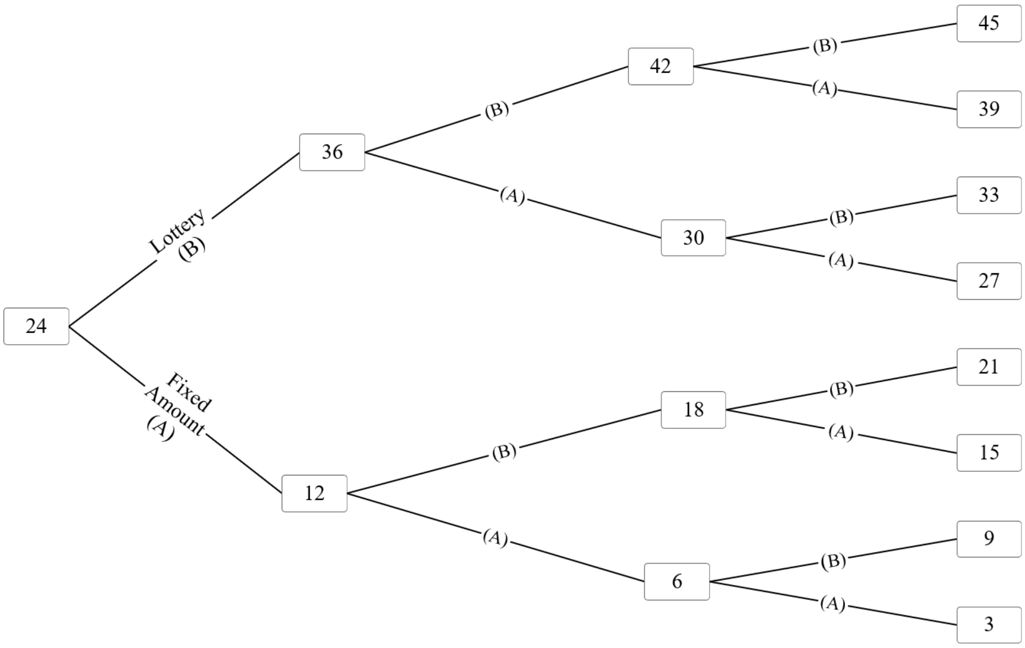

Notes: The staircase procedure following FA works as follows. First, each respondent was asked whether they would prefer to receive 24 EUR for sure or whether they preferred a 50:50 chance of receiving 45 EUR or nothing. In case the respondent opted for the fixed amount (A), the fixed amount being offered in the second question decreased to 12 EUR. If, on the other hand, the respondent opted for the lottery (B), the safe amount was increased to 36. Working further through the tree follows the same logic.

[1]Goethe University Frankfurt
[2]Leibniz Institute for Financial Research SAFE, Frankfurt
[3]University of Innsbruck

Figure A2: Effect of task-related incentives on risk-taking - Distributions

Panel A: Private investors

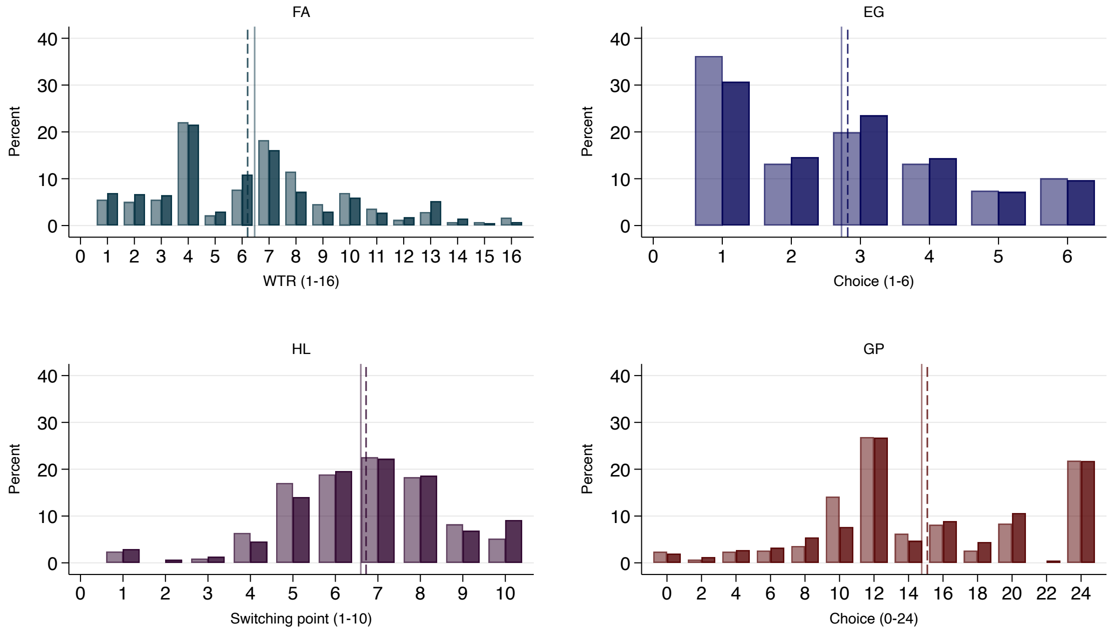

Panel B: Professional Investors

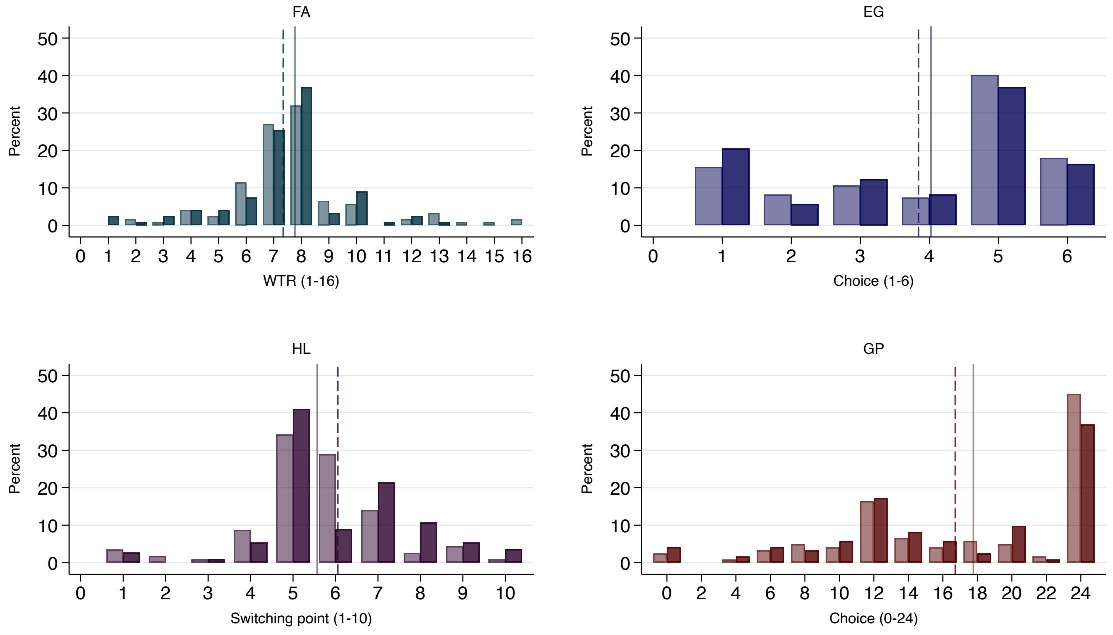

Figure A2 (continued): Effect of task-related incentives on risk-taking - Distributions

Panel C: Students

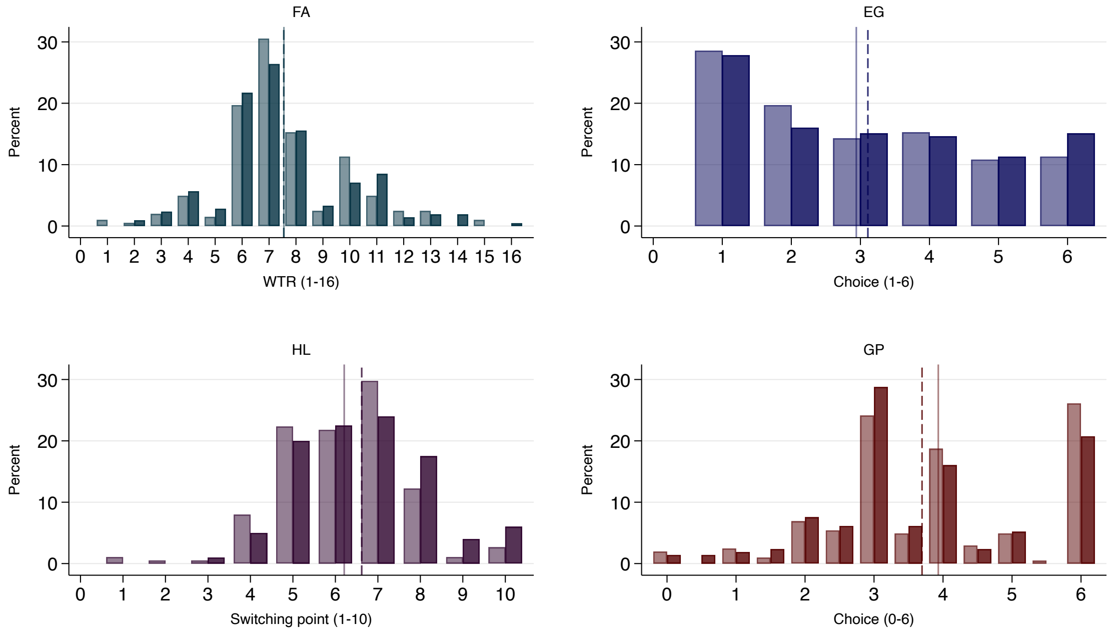

Notes: The figure shows the distributions of respondents' choices in the four experimental tasks (FA in emerald, EG in blue, HL in purple, and GP in maroon) by incentive condition, separately for each subject pool. The light (dark) shaded bars represent the choices of subjects in the FLAT (INCENTIVES) condition. The solid (dashed) line represents the mean in the FLAT (INCENTIVES) condition. In the FLAT condition, subjects receive a fixed participation reward only, amounting to €12 for private investors and professional investors, and to €3 for students. Subjects in the INCENTIVES condition *in addition* are paid the earnings resulting from their choice in one randomly determined experimental task. Panel A represents choices in the private investor sample. Panel B (C) represents choices in the professional investor (student) sample. FA takes a value between 1 and 16, according to the certainty equivalent resulting from the last of the four choices in the staircase risk task. EG is the rank (1-6) of the gamble chosen from a menu of six 50/50 gambles, increasing in risk. HL is the number of decision rows left after switching to the higher-risk lottery, ranging from 0 to 10. GP is the EUR amount invested in the risky project and takes values between 0 and 24 (0 and 6 for students). We report the p-values of Kolmogorov-Smirnov tests for equality of distributions in Table A1.

Figure A3: Task-related incentives and extreme choices

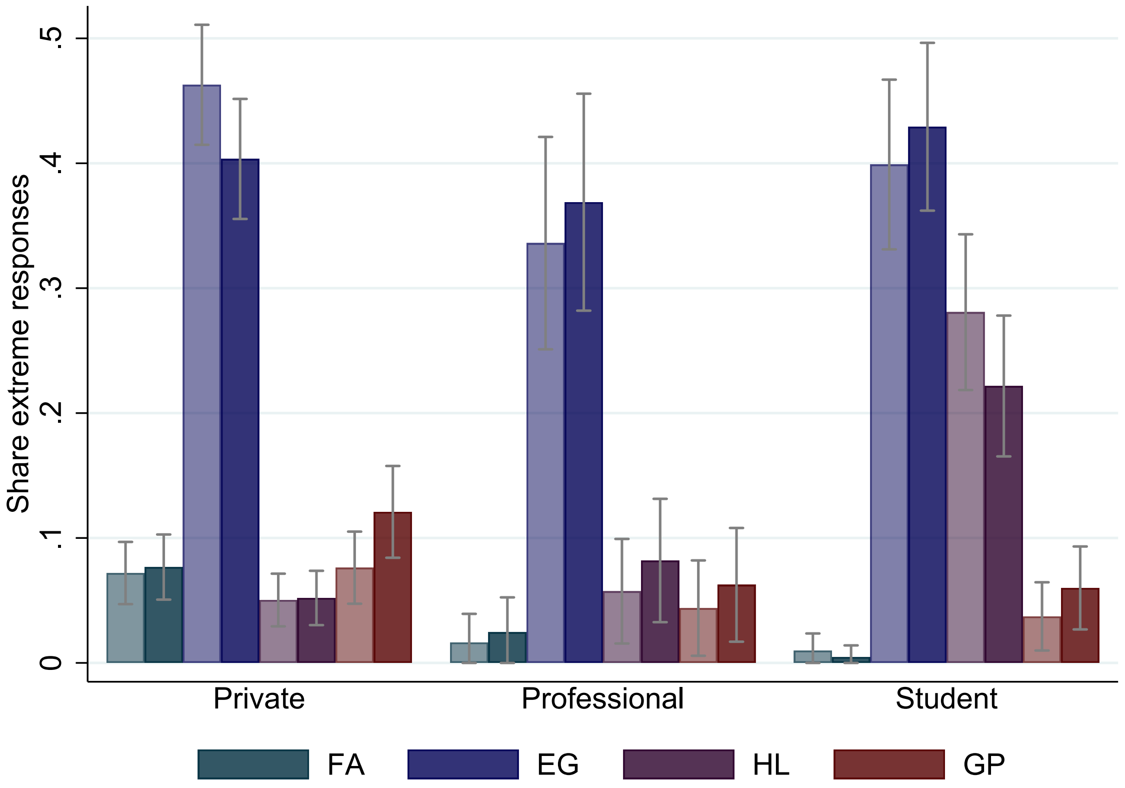

Notes: The figure shows the share of respondents who choose the least or the most risky option in the four experimental tasks (FA in emerald, EG in blue, HL in purple, and GP in maroon) by incentive condition, separately for the three subject pools. The light (dark) shaded bars represent the choices of subjects in the FLAT (INCENTIVES) condition. In the FLAT condition, subjects receive a fixed participation reward only, amounting to €12 for private investors and professional investors, and to €3 for students. Subjects in the INCENTIVES condition *in addition* are paid the earnings resulting from their choice in one randomly determined experimental task. The left block of bars represents probabilities of extreme choices in the private investor sample. The middle (right) block represents probabilities of extreme choices in the professional investor (student) sample. Error bars indicate 95%-confidence intervals. We report the p-values of two-sided t-tests for equality of mean choices in Table A4. Note that the higher share of extreme answers for EG compared to the other tasks is not surprising as there are only 6 possible choices. Hence, the 2 extreme answers account for 1/3 of the decision space.

Figure A4: Consistency in responses across tasks – excluding HL

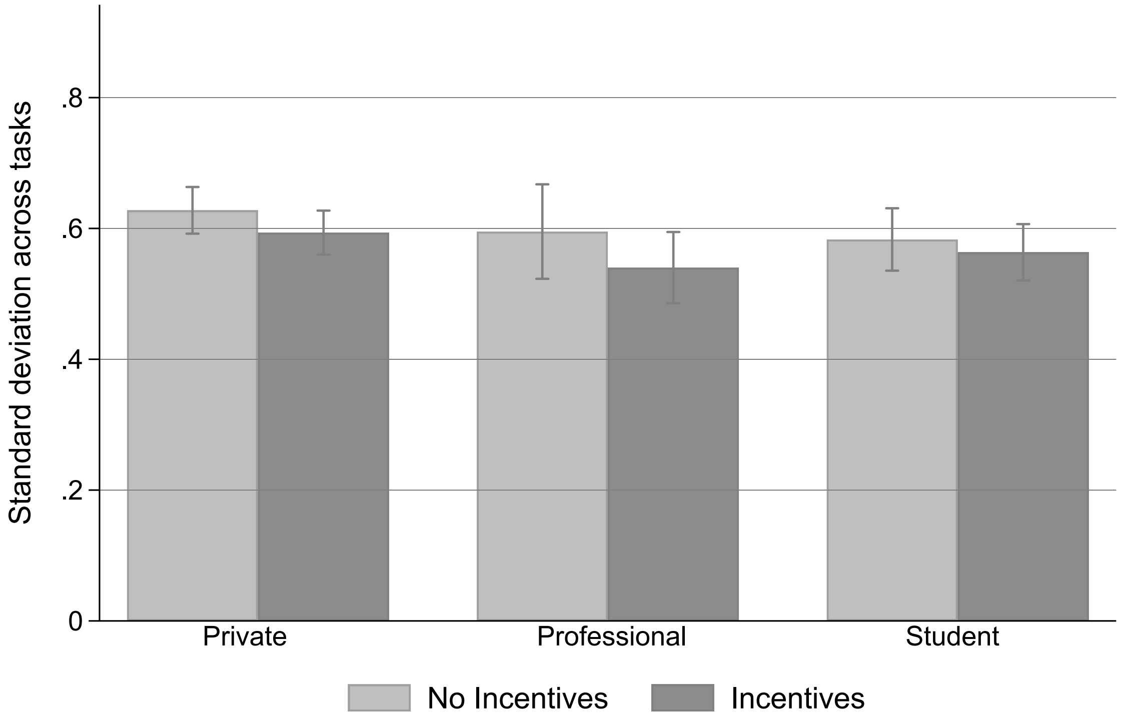

Notes: The figure compares the mean within-subject standard deviation for three of the four experimental tasks - excluding HL - by incentive condition separately for the three subject pools. We standardize choices in the single tasks by deducting the mean and dividing by the standard deviation of choices made in the given task in the relevant subject pool. We then calculate the within-subject standard deviation over a subject's three standardized choices. The light (dark) shaded bars refer to subjects in the FLAT (INCENTIVES) condition. In the FLAT condition, subjects receive a fixed participation reward only, amounting to €12 for private investors and professional investors, and to €3 for students. Subjects in the INCENTIVES condition *in addition* are paid the earnings resulting from their choice in one randomly determined experimental task. Error bars indicate 95%-confidence intervals.

Figure A5: Effect of task-related incentives on risk-taking - Student sample

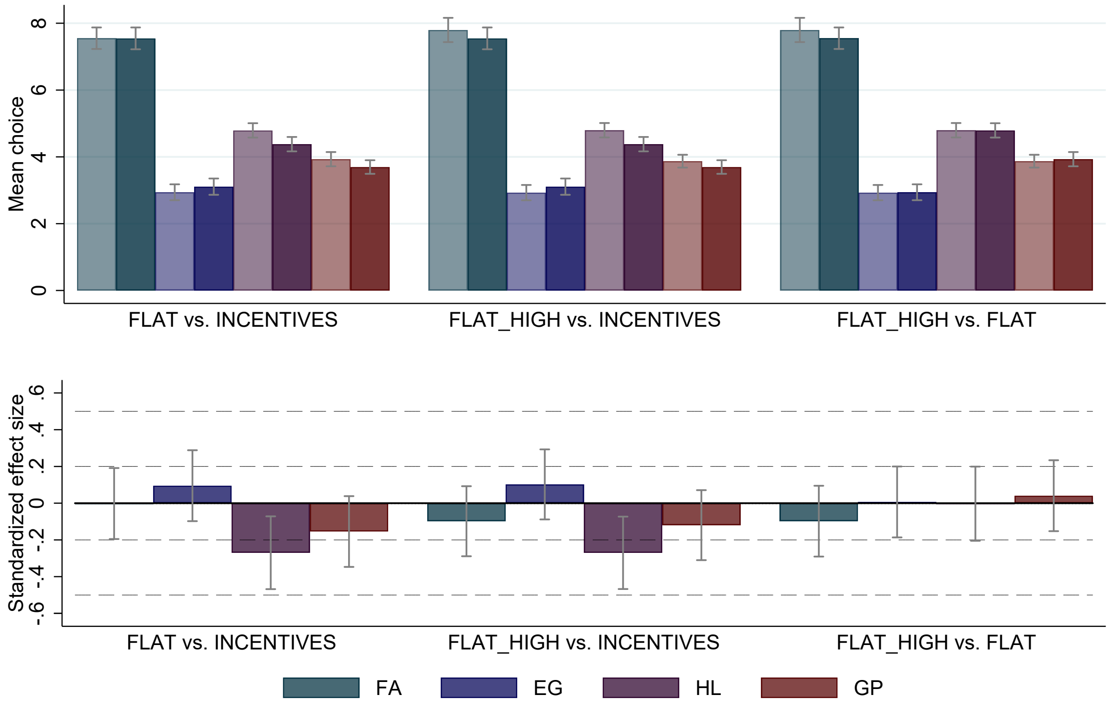

Notes: The upper panel shows pairwise comparisons of the mean choices in the four different tasks (FA in emerald, EG in blue, HL in purple, and GP in maroon) across subjects in the three different incentive conditions in the student subject pool. The left block compares subjects in the FLAT condition (light shaded bars) to subjects in the INCENTIVES condition (dark shaded bars). The middle block compares subjects in the FLAT_HIGH condition (light shaded bars) to subjects in the INCENTIVES condition (dark shaded bars). The right block compares subjects in the FLAT_HIGH condition (light shaded bars) to subjects in the FLAT condition (dark shaded bars). Subjects in the FLAT (FLAT_HIGH) condition receive a fixed participation fee of €3 (€9). Subjects in the INCENTIVES condition receive a fixed participation fee of €3 plus the earnings resulting from their choice in one randomly determined task. FA takes a value between 1 and 16, according to the certainty equivalent resulting from the last of the four choices in the staircase risk task. EG is the rank (1-6) of the gamble chosen from a menu of six 50/50 gambles, increasing in risk. HL is the number of decision rows left after switching to the higher-risk lottery, ranging from 0 to 10. GP is the EUR amount invested in the risky project and takes values between 0 and 6. Investment amounts can be adjusted in steps of €0.50. Higher values imply higher risk tolerance across all four tasks. The lower panel shows standardized treatment effects. We standardize the choices of student subjects in the four different task by subtracting the mean and dividing by the standard deviation of the distribution of choices in the subject pool. We then regress standardized choices on an indicator for whether a subject has been assigned to the comparison incentive condition (INCENTIVES for the left and middle blocks, FLAT for the right block). Error bars indicate 95%-confidence intervals.

B Additional Tables

Table A1: Effect of task-related incentives on risk-taking

Panel A. Private investors

	FA	EG	HL	GP
FLAT	6.47	2.73	4.40	14.76
INCENTIVES	6.21	2.82	4.27	15.09
Δ	-0.26	0.09	-0.12	0.33
t-test (p-value)	0.272	0.436	0.406	0.460
KS test (p-value)	0.177	0.525	0.956	0.583
Observations	821	821	634	821

Panel B. Professional investors

	FA	EG	HL	GP
FLAT	7.77	4.02	5.43	17.77
INCENTIVES	7.34	3.84	4.95	16.72
Δ	-0.43	-0.18	**-0.48**	-1.05
t-test (p-value)	0.135	0.425	0.032	0.237
KS test (p-value)	0.983	0.998	0.022	0.652
Observations	244	244	226	244

Panel C. Students

	FA	EG	HL	GP
FLAT	7.55	2.94	4.79	3.93
INCENTIVES	7.55	3.11	4.38	3.70
Δ	0.00	0.17	**-0.41**	-0.24
t-test (p-value)	0.984	0.333	0.008	0.116
KS test (p-value)	0.996	0.983	0.125	0.345
Observations	415	415	388	415

Notes: The table reports differences in risk-taking in the four different experimental task by incentive condition and subject pool. In the FLAT condition, subjects receive a fixed participation reward only, amounting to €12 for private investors and professional investors, and to €3 for students. Subjects in the INCENTIVES condition in addition are paid the earnings resulting from their choice in one randomly determined experimental task. Panels A, B and C show differences by incentive condition for the 821 subjects in the private investor sample, the 244 subjects in the professional investor sample, and the 638 respondents in the student sample, respectively. Mean differences that are significant at least at the 5 percent level are printed in bold. We report p-values of a two sided t-test of equal means and p-values of a Kolmogorov-Smirnov test of equal distributions.

Table A2: Standard deviation of choices

	All	Private investors			Professional investors			Students		
		INCENTIVES	FLAT	Difference	INCENTIVES	FLAT	Difference	INCENTIVES	FLAT	Difference
	(1)	(2)	(3)	(4)	(5)	(6)	(7)	(8)	(9)	(10)
Std. Dev.										
FA	3.02	3.45	3.32	-0.13	2.09	2.35	0.26	2.41	2.33	-0.08
				(0.45)			(0.20)			(0.61)
EG	1.75	1.63	1.69	0.06	1.80	1.73	-0.07	1.80	1.72	-0.08
				(0.46)			(0.67)			(0.52)
HL	1.77	1.95	1.82	-0.14	1.76	1.60	-0.16	1.54	1.49	-0.05
				(0.19)			(0.30)			(0.63)
GP	1.60	1.59	1.57	-0.02	1.76	1.69	-0.07	1.51	1.54	0.03
				(0.79)			(0.67)			(0.75)
Obs (FA/EG/GP)	1,480	417	404	821	122	122	244	203	212	415
Obs (HL)	1,248	328	306	634	114	112	226	188	200	388

Notes: The table reports standard deviations of the choices in the four different tasks, by incentive condition and subject pool. FA takes a value between 1 and 16, according to the ordinal rank of the certainty equivalent resulting from the last of the four choices in the staircase risk task. EG is the rank (1-6) of the gamble chosen from a menu of six 50/50 gambles, increasing in risk. HL is the number of decision rows left after switching to the higher-risk lottery, ranging from 0 to 10. GP is the euro amount invested in the risky project and takes values between 0 and 24 for private and professional investors, and values between 0 and 6 for students. For comparability, we align these values across samples by dividing the invested amount in the private and professional investor sample by 4.In the FLAT condition, subjects receive a fixed participation reward only, amounting to €12 for private investors and professional investors, and to €3 for students. Subjects in the INCENTIVES condition in addition are paid the earnings resulting from their choice in one randomly determined experimental task. We report p-values of an F-Test on the equality of standard deviations for each sample.

Table A3: Effort and consistency of choices across tasks

	**All**	**Private investors**			**Professional investors**			**Students**		
		INCENTIVES	FLAT	Difference	INCENTIVES	FLAT	Difference	INCENTIVES	FLAT	Difference
	(1)	(2)	(3)	(4)	(5)	(6)	(7)	(8)	(9)	(10)
Decision time										
FA	0.88	1.12	0.96	-0.16	0.91	0.82	-0.09	0.70	0.71	0.01
				(0.29)			(0.26)			(0.90)
EG	1.13	1.21	1.11	-0.09	1.87	1.37	-0.51**	0.91	0.99	0.09
				(0.31)			(0.01)			(0.42)
HL	2.58	2.72	3.12	0.41	3.79	3.18	-0.62	1.82	1.92	0.10
				(0.13)			(0.29)			(0.80)
GP	1.29	1.55	1.25	-0.31	2.00	1.87	-0.14	1.04	0.91	-0.12
				(0.19)			(0.72)			(0.17)
HL: multiple switchers	0.15	0.24	0.21	-0.03	0.08	0.07	-0.02	0.06	0.07	0.02
				(0.32)			(0.63)			(0.48)
Drop-out rate	0.08	0.12	0.10	-0.01	0.09	0.14	0.05	0.02	0.03	0.01
				(0.48)			(0.19)			(0.53)
Indiv. SD	0.78	0.79	0.77	-0.02	0.78	0.76	-0.02	0.80	0.75	-0.05
				(0.50)			(0.67)			(0.19)
Observations	1,480	404	417	821	122	122	244	212	203	415

Notes: The table reports differences in task-specific decision times in minutes, frequencies of multiple switching in the HL task, drop-out rates as well as the within-subject standard deviation of an individual's choices across the four tasks by incentive condition and subject pool. In the FLAT condition, subjects receive a fixed participation reward only, amounting to €12 for private investors and professional investors, and to €3 for students. Subjects in the INCENTIVES condition in addition are paid the earnings resulting from their choice in one randomly determined experimental task. Drop out rates are calculated based on the overall number of respondents who started the experiment (N = 1,661), of which 1,512 completed it. To calculate the within-subject standard deviation, we standardize subjects' choices in the four experimental tasks by subtracting the mean and dividing by the standard deviation of the distribution of choices in the respective task in the relevant subject pool. For each subject, we then calculate the standard deviation of the standardized choices in the four experimental tasks. We report p-values of two sided t-tests of equal means.

Table A4: Probability of extreme choices

	All	Private investors			Professional investors			Students		
		INCENTIVES	FLAT	Difference	INCENTIVES	FLAT	Difference	INCENTIVES	FLAT	Difference
	(1)	(2)	(3)	(4)	(5)	(6)	(7)	(8)	(9)	(10)
Extreme choice										
FA	0.04	0.08	0.07	-0.00	0.02	0.02	-0.01	0.00	0.01	0.01
				(0.79)			(0.65)			(0.54)
EG	0.41	0.40	0.46	0.06	0.37	0.34	-0.03	0.43	0.40	-0.03
				(0.09)			(0.59)			(0.53)
HL	0.07	0.12	0.08	-0.04	0.06	0.04	-0.02	0.06	0.04	-0.02
				(0.06)			(0.53)			(0.30)
GP	0.12	0.05	0.05	-0.00	0.08	0.06	-0.02	0.22	0.28	0.06
				(0.92)			(0.45)			(0.17)
Observations	1,480	404	417	821	122	122	244	212	203	415

Notes: The table reports differences in the propensity to choose the least or the most risky option in the four different tasks, by incentive condition and subject pool. In the FLAT condition, subjects receive a fixed participation reward only, amounting to €12 for private investors and professional investors, and to €3 for students. Subjects in the INCENTIVES condition in addition are paid the earnings resulting from their choice in one randomly determined experimental task. We report p-values of a two sided t-test of equal means for each sample.

Table A5: Descriptive statistics: Student sample with high flat fee

	ALL	INCENTIVES	FLAT_HIGH		
	Mean (SD)	Mean	Mean	P-value	Obs.
	(1)	(2)	(3)	(4)	(5)
Female	0.58 (0.49)	0.58	0.59	0.762	425
Age	24.11 (3.54)	24.16	24.06	0.546	425
Net income	738.13 (373.73)	727.14	749.28	0.8788	417
Stock investor	0.23 (0.42)	0.23	0.23	0.887	425
Smartphone	0.04 (0.18)	0.03	0.04	0.804	425
Total time	13.08 (6.87)	14.35	11.81	0.000	425
Payoff	8.34 (8.34) (3.30)	7.67	9.00	0.000	425

Notes: This table shows summary statistics for the 425 participants in the INCENTIVES versus FLAT_HIGH conditions in the student sample. Information of respondents' household net income is only available for 417 students. Stock investor is an indicator equal to one for participants who invest in stocks or stock mutual funds. Smartphone is an indicator of whether the respondent has participated in the experiment using a smartphone. Total time is the time (in minutes) a subject has spend to complete the entire experiment. Payoff is the final payoff participants receive after completing the experiment. It is fixed in the FLAT_HIGH condition. For subjects in the INCENTIVES condition, it depends on the choice and resulting outcome in one randomly determined experimental task. Task-related payoffs in the INCENTIVES condition are in addition to the fixed participation fee paid to subjects in the relevant FLAT_HIGH condition. Column 4 reports p-values from a two-sided t-test of equal means between subjects in the INCENTIVES and FLAT_HIGH condition.

Table A6: Order effects in student within-sample

	INCENTIVES			FLAT		
	Round 1 (1)	Round 2 (2)	Difference (3)	Round 1 (4)	Round 2 (5)	Difference (6)
Average choice						
FA	7.30	7.28	-0.02	7.41	7.36	-0.04
			(0.95)			(0.89)
EG	3.07	3.29	0.22	2.89	2.90	0.01
			(0.38)			(0.98)
HL	4.38	4.67	0.30	4.89	4.77	-0.12
			(0.21)			(0.61)
GP	3.53	3.70	0.17	3.91	3.82	-0.09
			(0.42)			(0.70)
Obs (FA/EG/GP)	110	103	213	103	110	213
Obs (HL)	106	98	204	91	104	195

Notes: The table reports average choices in the four different tasks, by incentive condition and wave. In the FLAT condition, subjects receive a fixed participation reward only, amounting to €12 for private investors and professional investors, and to €3 for students. Subjects in the INCENTIVES condition in addition are paid the earnings resulting from their choice in one randomly determined experimental task. We report p-values of a t-Test on the equality of means for each sample.

C Additional Analyses

Details on the power analysis

Our goal is to provide the basis for conclusive inference, including the case where we would not reject the null hypothesis of no effect of monetary incentivization. To this end, we seek to achieve a sufficiently high power for our statistical tests. In the absence of an indication of how large the effect of incentivization on average choices might be, we followed the reference points suggested by Cohen (1988) for behavioral sciences. As a lower bound for our analysis, we sought a probability of 90% to detect a 'small' effect size of less than 0.5 of a standard deviation.[4] This result corresponds to a sample size of at least $N = 85$ per incentive condition. For the most difficult to recruit sample, that of professional investors, we targeted a sample size of $N = 100$. For the samples of students and private investors, which are easier to recruit, we aimed at sample sizes that would allow us to detect even smaller effect sizes of $d = 0.33$ and $d = 0.20$ (the latter being the lowest threshold for power analysis as suggested by Cohen (1988)), respectively, rounding to targeted sample sizes of $N = 200$ and $N = 500$.

Table C1: Power analysis

	INCENTIVES VS FLAT
Private investors ($N_1 = 404$, $N_2 = 417$)	0.226
Professional investors ($N_1 = 122$, $N_2 = 122$)	0.415
Students ($N_1 = 212$, $N_2 = 203$)	0.318

Notes: The table provides an overview of the number of subjects in each incentive condition and reports the corresponding effect sizes (Cohen's d) we can detect with 90% power.

[4] Cohen (1988) argues that a 'medium' effect size of $d = 0.5$ is 'large enough to be visible to the naked eye'. To put effect sizes into the perspective of risk elicitation experiments, we refer to the extensive meta-study by Filippin and Crosetto (2016), who analyze the effect of gender on risk-taking. They find an average effect size of $d = 0.55$ for both the investment game and the gamble-choice task, and $d = 0.17$ for the multiple price list. However, while, some controversy persists on the effect of gender on risk-taking, the importance of incentivizing preference elicitation tasks seems to be almost universally accepted among experimental economists. We therefore believe that a threshold *below* the median effect of gender is a conservative benchmark for the presumed effect of incentivization.

Our average realized sample sizes per group were 410, 122, and 208 for private investors, professional investors, and students, respectively. These numbers are all well above the lower bound of $N = 85$ to detect effect sizes below $d = 0.5$ with 90% power. The corresponding *ex ante* effect sizes we are able to detect given our realized numbers of observations are summarized in Table C1.

D Instructions of the Online Experiment

Intro screen incentives treatment

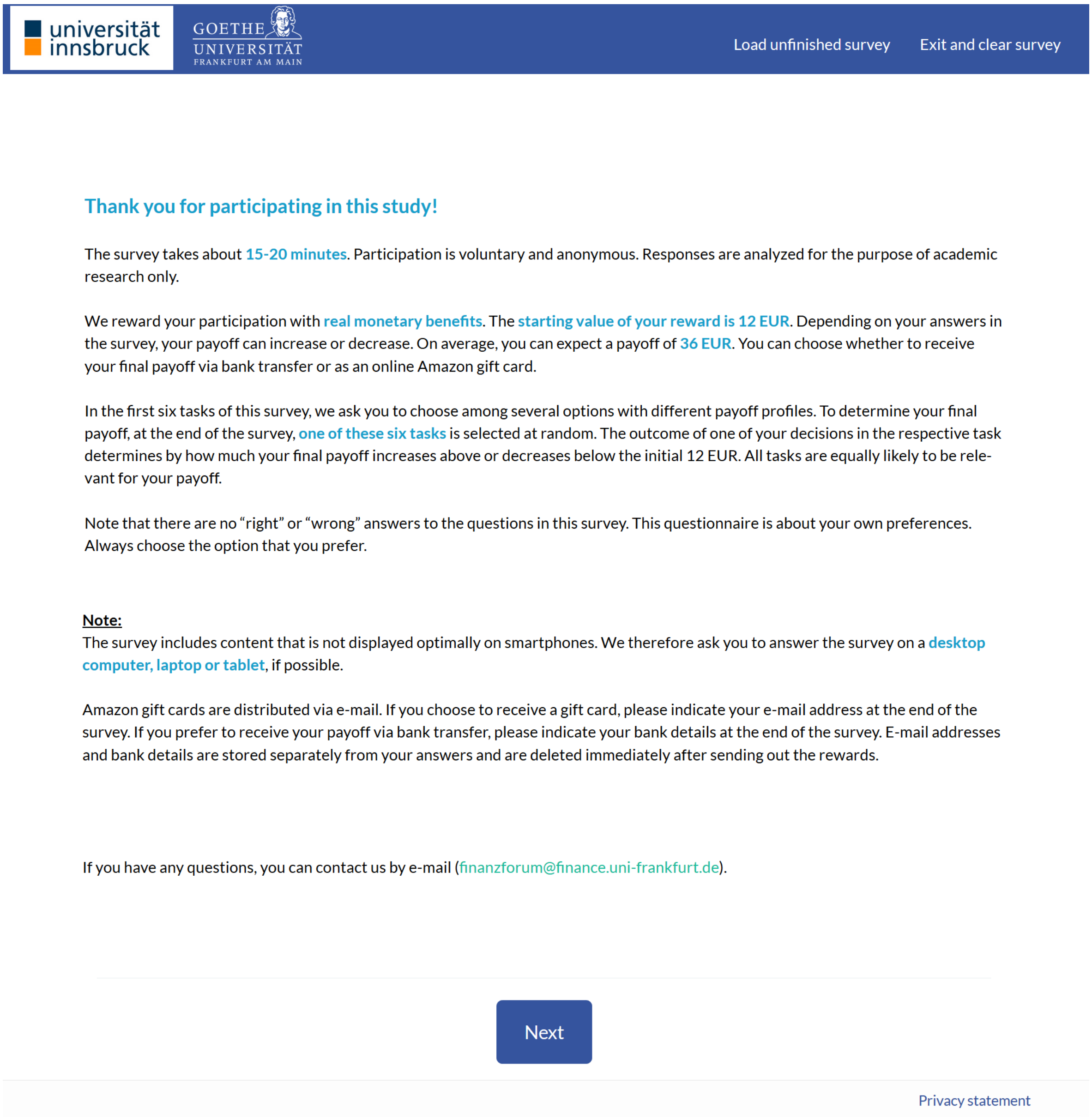

universität innsbruck

GOETHE UNIVERSITÄT FRANKFURT AM MAIN

Load unfinished survey

Exit and clear survey

Thank you for participating in this study!

The survey takes about **15-20 minutes**. Participation is voluntary and anonymous. Responses are analyzed for the purpose of academic research only.

We reward your participation with **real monetary benefits**. The **starting value of your reward is 12 EUR**. Depending on your answers in the survey, your payoff can increase or decrease. On average, you can expect a payoff of **36 EUR**. You can choose whether to receive your final payoff via bank transfer or as an online Amazon gift card.

In the first six tasks of this survey, we ask you to choose among several options with different payoff profiles. To determine your final payoff, at the end of the survey, **one of these six tasks** is selected at random. The outcome of one of your decisions in the respective task determines by how much your final payoff increases above or decreases below the initial 12 EUR. All tasks are equally likely to be relevant for your payoff.

Note that there are no “right” or “wrong” answers to the questions in this survey. This questionnaire is about your own preferences. Always choose the option that you prefer.

Note:
The survey includes content that is not displayed optimally on smartphones. We therefore ask you to answer the survey on a **desktop computer, laptop or tablet**, if possible.

Amazon gift cards are distributed via e-mail. If you choose to receive a gift card, please indicate your e-mail address at the end of the survey. If you prefer to receive your payoff via bank transfer, please indicate your bank details at the end of the survey. E-mail addresses and bank details are stored separately from your answers and are deleted immediately after sending out the rewards.

If you have any questions, you can contact us by e-mail (finanzforum@finance.uni-frankfurt.de).

Next

Privacy statement

Intro screen flat treatment

universität innsbruck GOETHE UNIVERSITÄT FRANKFURT AM MAIN

Load unfinished survey Exit and clear survey

Thank you for participating in this study!

The survey takes about **15-20 minutes**. Participation is voluntary and anonymous. Responses are analyzed for the purpose of academic research only.

We reward your participation with **real monetary benefits**. The **value of your reward is 12 EUR**. You can choose whether to receive your final payoff via bank transfer or as an online Amazon gift card.

In the first six tasks of this survey, we ask you to choose among several options with different hypothetical payoff profiles. Note that the payoff amounts that result from your decisions will not actually be paid out.

Note that there are no "right" or "wrong" answers to the questions in this survey. This questionnaire is about your own preferences. Always choose the option that you prefer.

Note:
The survey includes content that is not displayed optimally on smartphones. We therefore ask you to answer the survey on a **desktop computer, laptop or tablet**, if possible.

Amazon gift cards are distributed via e-mail. If you choose to receive a gift card, please indicate your e-mail address at the end of the survey. If you prefer to receive your payoff via bank transfer, please indicate your bank details at the end of the survey. E-mail addresses and bank details are stored separately from your answers and are deleted immediately after sending out the rewards.

If you have any questions, you can contact us by e-mail (finanzforum@finance.uni-frankfurt.de).

Next

Privacy statement

The *staircase procedure* by Falk et al. (2016, 2018) (FA)

Resume later | Exit and clear survey

Task: Decision sequence

Instructions

In the following **four decisions,** we ask you to choose between receiving a **fixed amount** (Option A) or participating in a **lottery** (Option B) which either pays **45 EUR** or **0 EUR** with equal probability (50/50).

The payoffs of the lottery are the same in each of the four decisions. The **fixed amount** is **different in each decision**.

If one of these four decisions is drawn at random to determine your payoff, your final earnings result as follows. If you choose to participate in the lottery, a fair coin is tossed to determine the outcome of the lottery. If the coin comes up "heads", your payoff increases by the positive amount, in addition to the initial payment. If the coin comes up "tails", you receive no additional payment. If you choose to receive the fixed amount, your payoff is equal to the fixed amount, in addition to the initial payment.

Next

universität innsbruck GOETHE UNIVERSITÄT FRANKFURT AM MAIN

Resume later Exit and clear survey

Task: Decision sequence

Decision **1** of 4

Which option do you prefer?

Option A: You receive a fixed amount of *24 EUR*.
Option B: You receive either *0 EUR or 45 EUR* with equal probability (50/50).

Option A: You receive a fixed amount of *24 EUR*.
Option B: You receive either *0 EUR or 45 EUR* with equal probability (50/50).

○ Option A: Fixed amount ○ Option B: Lottery

Next

universität innsbruck | GOETHE UNIVERSITÄT FRANKFURT AM MAIN | Resume later | Exit and clear survey

Task: Decision sequence

Decision **2** of 4

Which option do you prefer?

Option A: You receive a fixed amount of *36 EUR*.
Option B: You receive either *0 EUR or 45 EUR* with equal probability (50/50).

○ Option A: Fixed amount

○ Option B: Lottery

Next

universität innsbruck GOETHE UNIVERSITÄT FRANKFURT AM MAIN

Resume later Exit and clear survey

Task: Decision sequence

Decision **3** of 4

Which option do you prefer?

Option A: You receive a fixed amount of *42 EUR*.
Option B: You receive either *0 EUR or 45 EUR* with equal probability (50/50).

Option A: Fixed amount

Option B: Lottery

Next

Resume later   Exit and clear survey

Task: Decision sequence

Decision 4 of 4

Which option do you prefer?

Option A: You receive a fixed amount of *45 EUR*.
Option B: You receive either *0 EUR or 45 EUR* with equal probability (50/50).

○ Option A: Fixed amount   ○ Option B: Lottery

Next

The *gamble-choice task* by Eckel and Grossman (2002) (EG)

universität innsbruck GOETHE UNIVERSITÄT FRANKFURT AM MAIN

Resume later Exit and clear survey

Task: Coin toss

You are asked to choose your favorite option among the six options displayed in the table below.

Each option has two possible payoffs. A coin is tossed to determine which of the two amounts is paid out. Both payoffs hence result with equal probability.

Option	Coin comes up "heads" (payoff in EUR)	Coin comes up "tails" (payoff in EUR)
1	21 EUR	21 EUR
2	27 EUR	18 EUR
3	33 EUR	15 EUR
4	39 EUR	12 EUR
5	45 EUR	9 EUR
6	52 EUR	2 EUR

Please choose **your preferred option**.

If this decision is drawn at random to determine your payoff, your final earnings result as follows. To determine which of two amounts is paid out, a fair coin is tossed. If the coin comes up "heads", your payoff increases by the higher amount, in addition to the initial payment. If the coin comes up "tails", your payoff increases by the lower amount, in addition to the initial payment.

- **Option 1** [Heads: 21 EUR or Tails: 21 EUR]
- **Option 2** [Heads: 27 EUR or Tails: 18 EUR]
- **Option 3** [Heads: 33 EUR or Tails: 15 EUR]
- **Option 4** [Heads: 39 EUR or Tails: 12 EUR]
- **Option 5** [Heads: 45 EUR or Tails: 9 EUR]
- **Option 6** [Heads: 52 EUR or Tails: 2 EUR]

Next

The *paired lottery choice task* by Holt and Laury (2002) (HL)

universität innsbruck | GOETHE UNIVERSITÄT FRANKFURT AM MAIN | Resume later | Exit and clear survey

Task: Probability scenarios

You are asked to choose between two options, Option A and Option B, in each of the 10 scenarios below.

- Option A is a lottery that pays either 24.00 EUR or 19.20 EUR.
- Option B is a lottery that pays either 46.20 EUR or 1.20 EUR.

Starting from scenario 1, the **chances of the higher payoff increase** in each subsequent scenario as you move down the table.

	Option A				Option B			
			or:				*or:*	
	Probability	Payoff	Probability	Payoff	Probability	Payoff	Probability	Payoff
(1)	10%	24.00 EUR	90%	19.20 EUR	10%	46.20 EUR	90%	1.20 EUR
(2)	20%	24.00 EUR	80%	19.20 EUR	20%	46.20 EUR	80%	1.20 EUR
(3)	30%	24.00 EUR	70%	19.20 EUR	30%	46.20 EUR	70%	1.20 EUR
(4)	40%	24.00 EUR	60%	19.20 EUR	40%	46.20 EUR	60%	1.20 EUR
(5)	50%	24.00 EUR	50%	19.20 EUR	50%	46.20 EUR	50%	1.20 EUR
(6)	60%	24.00 EUR	40%	19.20 EUR	60%	46.20 EUR	40%	1.20 EUR
(7)	70%	24.00 EUR	30%	19.20 EUR	70%	46.20 EUR	30%	1.20 EUR
(8)	80%	24.00 EUR	20%	19.20 EUR	80%	46.20 EUR	20%	1.20 EUR
(9)	90%	24.00 EUR	10%	19.20 EUR	90%	46.20 EUR	10%	1.20 EUR
(10)	100%	24.00 EUR	0%	19.20 EUR	100%	46.20 EUR	0%	1.20 EUR

Please indicate whether you prefer **Option A or Option B** in each of the 10 scenarios.

If this decision is drawn at random to determine your payoff, your final earnings result as follows. In a first step, one of the 10 scenarios is selected at random. To determine the outcome of the option you selected for this scenario, one ball is drawn at random from an urn containing 100 blue and red balls. The proportion of blue and red balls corresponds to the probabilities of respective scenario (e.g., 10 blue and 90 red balls for Scenario 1). If a blue (red) ball is drawn, your payoff increases by the higher (lower) amount, in addition to the initial payment.

	Option A	Option B	
24.00 EUR with 10% probability **or** 19.20 EUR with 90% probability			46.20 EUR with 10% probability **or** 1.20 EUR with 90% probability
24.00 EUR with 20% probability **or** 19.20 EUR with 80% probability			46.20 EUR with 20% probability **or** 1.20 EUR with 80% probability
24.00 EUR with 30% probability **or** 19.20 EUR with 70% probability			46.20 EUR with 30% probability **or** 1.20 EUR with 70% probability
24.00 EUR with 40% probability **or** 19.20 EUR with 60% probability			46.20 EUR with 40% probability **or** 1.20 EUR with 60% probability
24.00 EUR with 50% probability **or** 19.20 EUR with 50% probability			46.20 EUR with 50% probability **or** 1.20 EUR with 50% probability
24.00 EUR with 60% probability **or** 19.20 EUR with 40% probability			46.20 EUR with 60% probability **or** 1.20 EUR with 40% probability
24.00 EUR with 70% probability **or** 19.20 EUR with 30% probability			46.20 EUR with 70% probability **or** 1.20 EUR with 30% probability
24.00 EUR with 80% probability **or** 19.20 EUR with 20% probability			46.20 EUR with 80% probability **or** 1.20 EUR with 20% probability
24.00 EUR with 90% probability **or** 19.20 EUR with 10% probability			46.20 EUR with 90% probability **or** 1.20 EUR with 10% probability
24.00 EUR with 100% probability			46.20 EUR with 100% probability

Next

The *investment game* by Gneezy and Potters (1997) (GP)

universität innsbruck GOETHE UNIVERSITÄT FRANKFURT AM MAIN

Resume later Exit and clear survey

Task: Project investment decision

You receive 24 EUR. You are asked to decide how much of this amount you want to invest in a project.

With a probability of 50%, the project is successful and pays 2.50 EUR for each Euro invested. With equal probabilty of 50%, the project is not successful and you lose the amount you invested.

You can adjust the amount you wish to invest in steps of 2 EUR.

Those EUR you do not invest are yours to keep.

Here are the payoff rules again:

Probability	Project outcome	Your payoff (EUR)
50%	Success	***amount kept*** ***+ 2.5 x amount invested***
50%	No success	***amount kept***

Please indicate **how many EUR** you wish to **invest** in the project.

If this decision is drawn at random to determine your payoff, your final earnings result as follows. To determine if your investment is a success, a fair coin is tossed. If the coin comes up “heads”, the project is successful. If the coin comes up “tails”, the project is not successful. The amount by which your final payoff increases in addition to the initial payment hence depends on how much you choose to invest in the project, and on whether or not the project is successful.

0 EUR 2 EUR 4 EUR 6 EUR 8 EUR 10 EUR 12 EUR 14 EUR 16 EUR 18 EUR 20 EUR 22 EUR 24 EUR

Next
